# Fractionation of Squid Pens with Ionic Liquids—An Upgraded β-Chitin and Shellfish Protein Production

**DOI:** 10.1021/acssuschemeng.4c04217

**Published:** 2025-02-12

**Authors:** Pedro Y. S. Nakasu, Vinicius Piccoli, Antonio Ovejero-Pérez, Priyanka Kumar, Amir Al Ghatta, Susiana Melanie, Cariny Polesca, Leandro Martinez, Jason P. Hallett

**Affiliations:** † Department of Chemical Engineering, Imperial College London, SW7 2AZ London, U.K.; ‡ Department of Materials, Imperial College London, SW7 2AZ London, U.K.; § Department of Physical Chemistry, Institute of Chemistry, Universidade Estadual de Campinas (UNICAMP), CEP 13083-862 Campinas, Brazil; ∥ CICECO—Aveiro Institute of Materials, Department of Chemistry, University of Aveiro, 3810-193 Aveiro, Portugal

**Keywords:** process design, squid waste, technoeconomic analysis, molecular dynamics, alternative protein

## Abstract

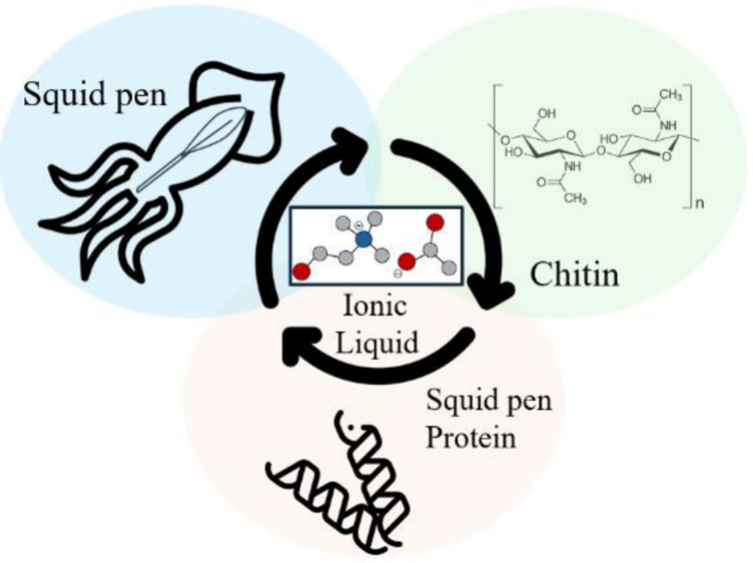

This study investigates the utilization of squid pen waste through a biocompatible ionic liquid approach, focusing on choline acetate, [Ch][OAc]. This ionic liquid effectively extracts over 80 wt % of protein from squid pen waste. To optimize the extraction process, a factorial design of experiments was employed to achieve a protein recovery of 75% at an estimated purity of 86%, along with highly acetylated, crystalline β-chitin with a purity of up to 95%. The extracted protein was subsequently used to create biocomposite films from α- and β-chitosan, demonstrating impressive tensile strengths of 93.15 ± 7.9 and 83.5 ± 6.2 MPa, respectively, while maintaining hydrophilic properties (θ_water_ < 90°). Molecular dynamics simulations revealed that the anion [OAc]^−^ exhibits a stronger affinity for protein surfaces compared to other anions, while its combination with the cation [Ch]^+^ optimally facilitates protein recovery. A material mass balance indicated that from 1 kg of dry squid pen, 0.526 kg of protein and 0.34 kg of chitin were recovered. However, high solvent usage significantly impacts energy demands and CO_2_ emissions, generating approximately 4.27 kg of CO_2_ per kg of product, with 61% attributed to protein production. Technoeconomic analysis demonstrated that solvent costs account for nearly 65% of the minimum selling price of the protein, estimated at $9 kg^–1^, which decreases to $0.6 for each kilogram of coproduced β-chitin. Technoeconomic analysis showed that solvent costs comprise nearly 65% of the minimum selling price of the protein, which can reach $9 kg^–1^, but this price decreases to $0.6 for each kilogram of coproduced β-chitin. This research underscores the potential of squid pen waste as a valuable resource while highlighting the need for sustainable solvent management strategies.

## Abstract 2

Ionic liquids can help to build a shellfish biorefinery by effectively fractionating its components.

## 1 Introduction

Seafood is a highly traded global food commodity, and it encompasses a wide range of species, including freshwater and marine finfish, bivalves, decapods, cephalopods, algae, and cyanobacteria.^1^ In 2020, global seafood production reached 214 million tons, with the squid fishery representing about 4.3% of all marine catches by volume and about 7% by value worldwide.^1,2^ The Food and Agricultural Organization of the United Nations estimates the seafood trade to be worth U.S. $151 billion, with a projected 13% growth in production value by 2030.^1^ Seafood categories are generally considered to have better environmental performance compared to other protein-rich foods, particularly in terms of sustainability.^3^ However, the seafood supply faces various challenges, including increasing demand due to overfishing, species depletion, environmental pollution, global warming, and biodiversity changes that ultimately have impacted the squid population over the last decades.^4^

Interest in the biorefining of seafood waste through environmentally friendly processing is a result of a growing understanding of how traditional fishing processes affect the environment and the stringent pollution control requirements imposed by regulatory bodies.^3,5^ Squids differ from fish in their high growth rate, short life span, and high feeding behavior. These characteristics end up exerting a high predation pressure on zooplankton, fish, and other squid prey, as in the case of the invasive Humboldt squid in the eastern North Pacific.^4,6^ There is then a necessity for population control and, of course, the introduction of biorefinery strategies for completely sustainable consumption and utilization of squid waste.^7^

Large amounts of squid waste are produced from wild-caught fisheries. It is estimated that more than 40% of the total body weight of squid ends up as processing byproducts, including the viscera, pens, and skins.^8,9^ These byproducts are routinely dumped in landfills or released into bodies of water. Incineration, composting, and anaerobic digestion could be an alternative, but only in more developed countries such as the U.K. and Australia, with disposal costs up to U.S. $150 per ton.^10^ This introduces demand for the implementation of efficient and cutting-edge treatment and utilization methods to minimize any adverse environmental effects on aquatic ecosystems or land and to encourage more productive practices that minimize biomass, energy, or nutrient losses.^1^

The squid pen, or gladius, is an internalized shell that acts as both a point of attachment for significant muscle groups and a barrier to protect the visceral organs.^11^ The pen’s distinctive protein and β-chitin composition is what gives it its strength and flexibility. The weight distribution of the pens from different species of squid is 25–49% β-chitin, 43–75% protein, and relatively low ash content.^11^ A protein layer surrounds the β-chitin nanofibrils, which are arranged parallel to the pen’s long axis by α-helical protein coils.^11,12^

Squid pens valorization has been focused on obtaining protein hydrolysates—via alkaline or enzymatic hydrolysis—with antioxidant properties or as fermentation media for microorganisms.^7,11^ These methods, however, do not entail the isolation of the squid protein to obtain a high-purity protein isolate, as the protein recovery methods such as dialysis or membrane separation, would be costly.^13^ Squid pen utilization for β-chitosan production usually employs harsh chemicals such as hydrochloric acid and sodium hydroxide^14^ and generates high volumes of wastewater, which negatively impacts the water footprint of the process.^15^ Obtaining a solid protein stream would therefore facilitate protein transport and storage.

Ionic liquids (ILs) are liquid salts at room temperature that present interesting physicochemical properties, such as high vapor pressure, conductivity, and high thermal stability. They can be tailored for different purposes by choosing different cation and anion combinations.^16,17^ There has been a debate on the “green-ness” of ILs due principally to their eco-toxicity^18^ (i.e., how it negatively impacts the growth of microorganisms, plants, or animal tissues), but this depends on the IL and process configuration. If the IL is fully recovered and the processing water streams are also recycled, then, a greener process can be ensured.^19^ Literature studies entailed solubilizing chitin and chitosan with ILs for energy, pharmaceutical, and medical applications, but not the fractionation of shellfish waste. A study by Shamshina and Abidi^20^ has shown that acidic ILs such as 1-butyl-3-methylimidazolium hydrogen sulfate, [BMIM][HSO_4_], can efficiently fractionate shrimp shells to produce high-purity chitin. Shrimp shells present more complex composition such as calcium carbonate (20–50 wt %), proteins (20–40 wt %), α-chitin (15–40 wt %), and lipids (0–14 wt %).^21^ However, there are no studies on the holistic biorefinery utilization of squid waste with ILs.

In this study, we aim to explore the use of ILs in the fractionation of squid pens to produce high-purity β-chitin and protein as two solid streams. We investigated the influence of the structure of the anion and cation of the IL on protein extraction in an IL screening. Once the IL was chosen, optimization of the fractionation parameters determined the best conditions to ensure a high protein yield. Then, recycling of the IL under a limited number of batches was probed to ensure that the IL performance was retained along the cycles. Characterization of the produced β-chitin and protein confirmed the efficiency of fractionation. Biocomposite chitosan and squid protein films were produced to underpin the potential utilization of the protein in biomaterials. A mass balance of the fractionation process, together with a technoeconomic analysis, indicated the process’s feasibility. Lastly, molecular dynamic (MD) simulation helped us to theorize the explanation of the choline acetate selectivity toward protein extraction and further isolation.

## 2 Materials and Methods

### 2.1 Feedstock and Reagents

All experiments used European-caught (*Loligo* sp.) squid pens sourced from a fish market in Spain and stored at −80 °C. Before fractionation, the different batches of squid pens were defrosted, washed in deionized (DI) water, rinsed with ethanol, cut into smaller pieces, and dried at 40 °C overnight (Figure S1 in the Supporting Information (SI)). Then, they were blended into a powder with a coffee grinder (Dr. Mills, San Jose, CA, USA) and sieved to a particle size between 180 and 850 μm. A sampling procedure similar to the cone and quartering mentioned by Venables and Wells^22^ where the homogenized sample was divided into smaller quarters, and when approximately 100 g of sample (from 1 kg) was obtained, it was separated for the experiments. Chemicals were sourced from Sigma-Aldrich, U.K., and included monoethanolamine (MEA, ≥98%), methanesulfonic acid (MeSO_3_H, ≥99%), glacial acetic acid (HOAc, 100%), choline bicarbonate ([Ch][HCO_3_], 80 wt %), sulfuric acid (H_2_SO_4_, 66.3 wt %), sodium bisulfate (NaHSO_4_, >90%), sodium acetate (NaOAc, ≥99%), and sodium hydroxide (NaOH, ≥97%).

### 2.2 Squid Pen Chemical Characterization

Squid pens were characterized in a similar alkaline extraction procedure by Chaussard and Domard.^39^ Briefly, the extractive content of squid pens was determined by two consecutive 12 h Soxhlet extractions with ethanol and then cyclohexane. Then, the squid pens were extracted with NaOH 1 mol·L^–1^ at 80 °C for 3 h for quantitative protein removal; the dry β-chitin mass was considered protein-free after CHN analysis established that its nitrogen content was 6.90%. The ash content of the squid pen samples was determined after ashing the dry squid pens in a muffle oven for 6 h at 600 °C.

### 2.3 IL Synthesis and Characterization

Six ILs were synthesized by dropwise addition of equimolar amounts of the acids (acetic, methanesulfonic, or sulfuric) to the base (choline bicarbonate or monoethanolamine) under stirring and cooling with an ice bath (0 °C). The six ILs were: choline hydrogen sulfate, [Ch][HSO_4_], choline methanesulfonate, [Ch][MeSO_3_], choline acetate, [Ch][OAc], monoethanolammonium hydrogen sulfate, [MEA][HSO_4_], monoethanolammonium methanesulfonate, [MEA][MeSO_3_], and monoethanolammonium acetate, [MEA][OAc]. Once the addition finished, the synthesized IL was stirred for 1 h and had its acid–base ratio and water content adjusted to 1:1 and 20 wt %, respectively. IL water content was measured using a V20 Volumetric Karl Fischer Titrator (Mettler-Toledo). Acid–base ratios of [Ch][HSO_4_] and [MEA][HSO_4_] were determined by titration with 0.1 M NaOH, using a G20S Compact Titrator (Mettler-Toledo, Columbus, USA), using potassium hydrogen phthalate (Sigma-Aldrich, U.K.) as a primary standard.

### 2.4 IL Screening Assays

Briefly, 9.0 g of the IL with 20 wt % water content was added to 1.0 g of squid pen powder in a flat-bottom glass vial. The vials were then heated to the target temperature, 100 °C, in a hot plate with constant stirring at 300 rpm for 3 h in a procedure similar to that employed by Polesca et al.^23^ The vials were then cooled to room temperature, and then 25 mL of ethanol was added. The slurry was filtered, and the filtered solid β-chitin, the retentate, was washed three times with 5 mL water portions and air-dried (Figure S1 in the SI). The filtrate had its ethanol content evaporated in a rotary evaporator at 40 °C and 50 mbar. Approximately 10 g of deionized (DI) water was added to precipitate the protein from the filtrate and the IL solution; the mixture was left sitting for 30 min, and it was centrifuged at 1500*g* for 10 min. The protein was washed thrice with 10 mL DI water and freeze-dried (FreeZone 6, Labconco, MO, USA) to yield a white powder (Figure S1 in the SI). β-Chitin and protein purity were estimated by CHN analysis (Medac Ltd., U.K.), and the calculations were detailed in Section 3. Three control reactions were also performed with NaHSO_4_ (1 mol·L^–1^, pH = 1.8), NaOAc (1 mol·L^–1^, pH = 7.0) and NaOH (1 mol·L^–1^, pH = 13), at the same conditions, 100 °C, 3 h at 300 rpm to evaluate the effect of pH in the protein extractions.

### 2.5 Optimization of Protein Extraction for [Ch][OAc]

Once [Ch][OAc] was chosen as the best IL for protein extraction, the optimization of the extraction parameters consisted of three series of experiments: (1) design of experiments (DoE): a 2^3^ + 3 (center points) factorial design; (2) single-variable experiments for water content and extraction time. The main goal of these experiments was to attain a high yield and purity of the squid protein while also producing a high-purity β-chitin. A complete description of the experiments can be found in the SI (Section S3.1).

### 2.6 Recycling of [Ch][OAc]

**Figure 1:**
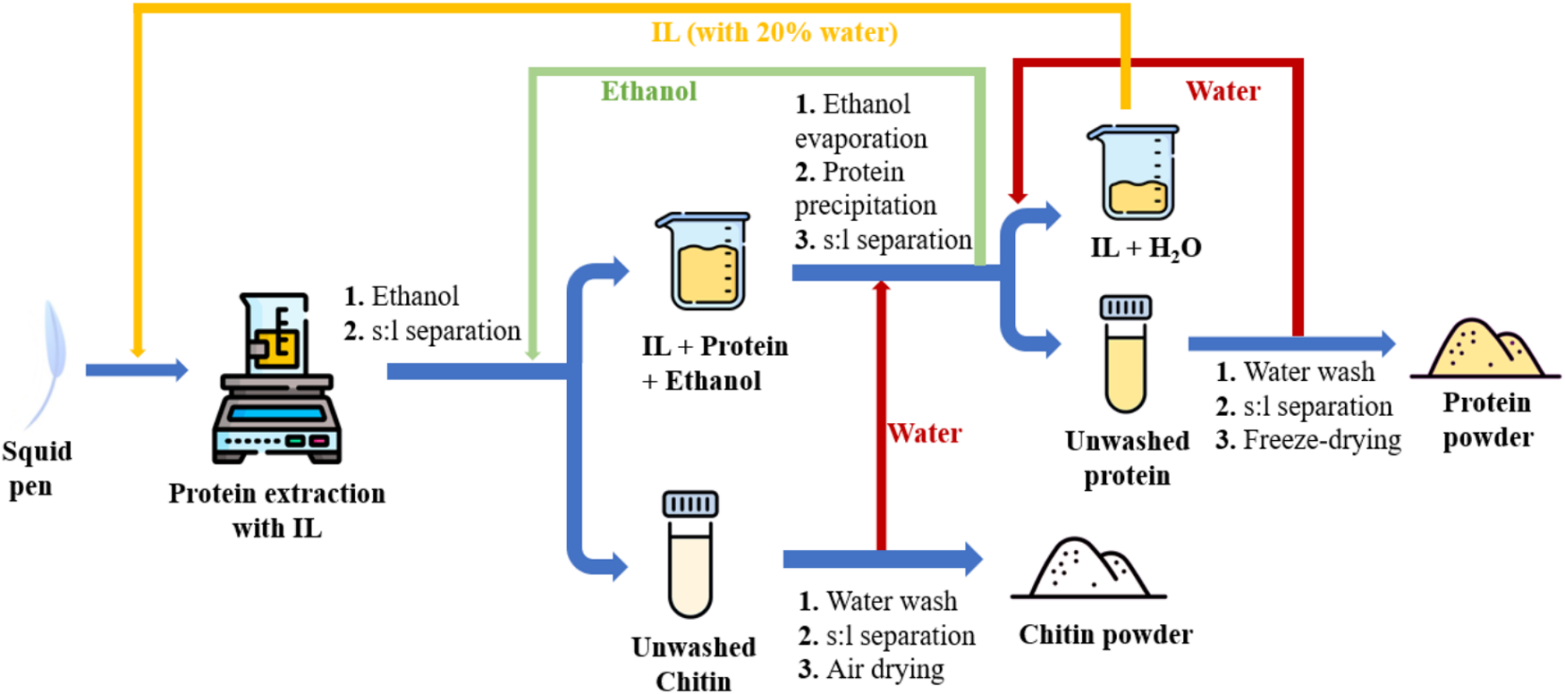
Recycling of [Ch][OAc] used for squid pen protein extraction.

Once protein extraction had been optimized, an IL recycling experiment was performed. The protein extraction was performed at 100 °C, 5 wt % solids loading, 20 wt % water content, and 2 h. The main difference from the previous experiments was that the wash water fractions from the β-chitin and protein were used to precipitate the protein and then added back to the aqueous IL, respectively (Figure 1). The IL underwent recycling for five iterations, totaling six cycles in all.

### 2.7 Biocomposite Films Production

The chitosan-squid pen protein films were produced by solution casting in silicone molds. The casting solutions were prepared by mixing two solutions; the first one contained 5 wt % of chitosan (α or β) in a 3 wt % acetic acid solution and the second one contained 2.5 wt % squid pen protein and 0.75 wt % glycerol in a 3 wt % acetic acid solution with a final proportion by mass of 6.7/3.3/1.0 of chitosan/protein/glycerol. After being cast, the molds were dried in a convection oven at 50 °C overnight. The films were then carefully removed from the molds and cut into strips of 1 × 5 cm^2^ and characterized by Fourier transform infrared (FT-IR), mechanical testing, thickness, and contact angle (Section S4.2).

### 2.8 Material Characterization—Analytical Methods

Once the β-chitin and protein were obtained under the optimized conditions with [Ch][OAc]—100 °C, 2 h, 5 wt % solids loading, and 20 wt % water—a comprehensive material characterization was performed to better understand the two streams that were produced. Details regarding the statistical analyses and analytical procedures for the characterization techniques ^1^H NMR, FT-IR, thermogravimetric analysis (TGA), scanning electron microscopy (SEM), X-ray diffraction (XRD), sodium dodecyl sulfate-polyacrylamide gel electrophoresis (SDS-PAGE), CHN analysis, and amino acid profiling can be found in SI (Section S4.2).

### 2.9 MD Simulations

Equilibrium molecular dynamics simulations were conducted utilizing GROMACS 2018.3 CUDA^24,25^ to explore the enhanced efficiency of protein extraction from squid pen by ILs based on acetate and choline. The model protein used was ubiquitin. Initial systems were prepared with 3.0 mol·L^–1^ IL aqueous solution boxes generated via Packmol.^26,27^ These systems comprised ILs formed by the combination of choline ([Ch]^+^), monoethanolamonium ([MEA]^+^), acetate ([OAc]^−^), and methanesulfonate ([MeSO_3_]^−^) ions. Additional information regarding the simulations can be found in the SI (Section S7).

### 2.10 Technoeconomic Analysis

Aspen Plus version 11 was employed for process simulation to estimate economic costs and the environmental impact of removing ethanol and water from the IL for its reuse. This was done following similar methodologies reported previously.^23,28^ Additional information regarding the simulations can be found in the SI (Section S8).

## 3 Results and Discussion

### 3.1 Chemical Composition of Squid Pen

The chemical composition of the squid pens on a dry basis is 30 ± 1.3 wt % chitin, 68.6 ± 2.6 wt % protein, 0.5 ± 0.02 wt % ash, and 1.1 ± 0.26 wt % lipids. These values are quite similar to those obtained for the composition of *Illex argentinus*’ squid pen by Susana Cortizo et al.^29^ with 31 wt % of chitin, 64 wt % of protein, 1.0 wt % ash, and 2.3 wt % lipids. Additionally, Kurita et al.^30^ also found similar values for the squid pens of *Ommastrephes bartrami*, 35–40 wt % chitin, and 58 wt % of protein. These values suggest that squid pens are relatively simple shellfish waste due to their composition, consisting mainly of proteins and chitin.

### 3.2 IL Screening for Protein Extraction

**Figure 2:**
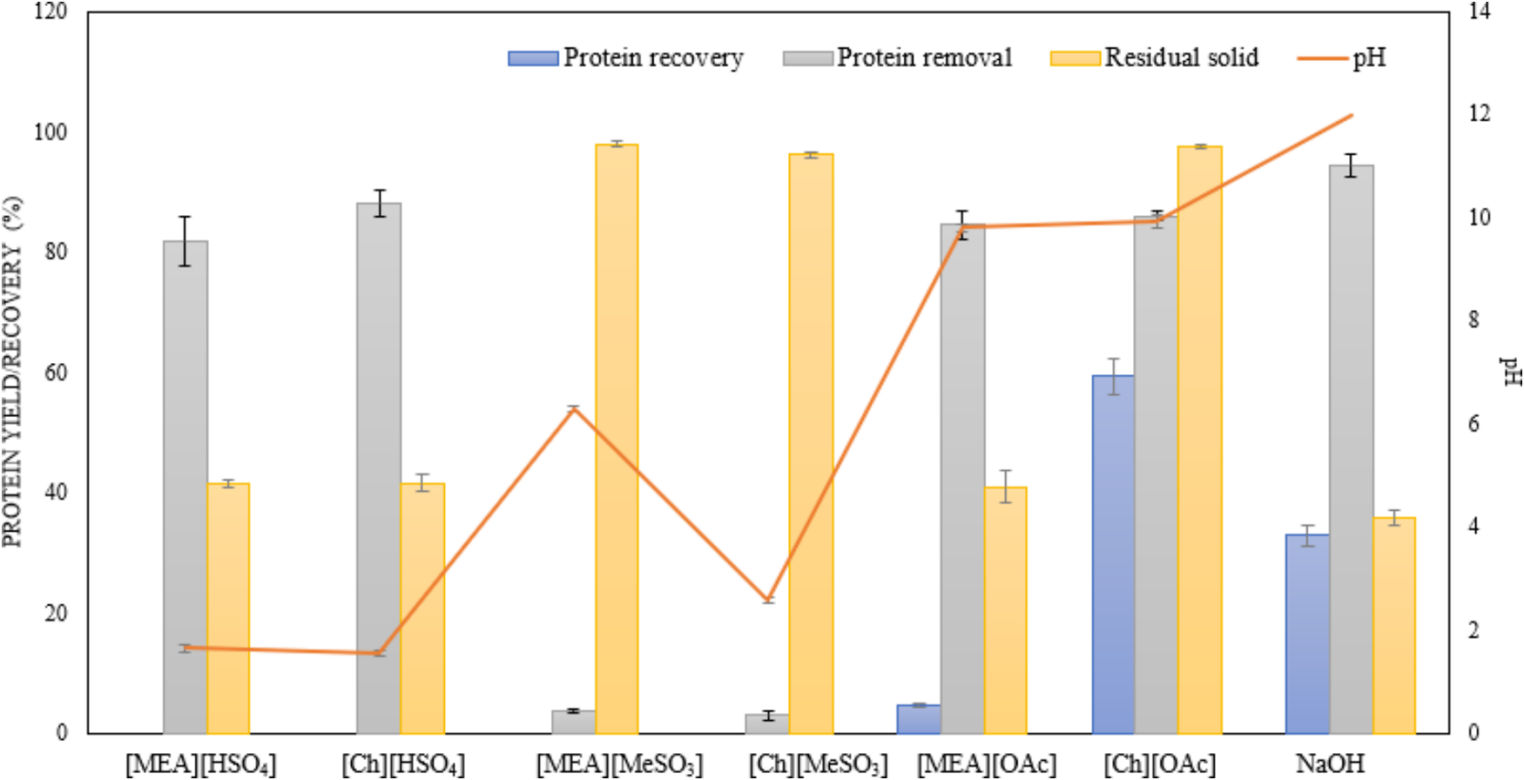
IL screening of squid protein extraction: protein removal, recovery, and pH of the IL solution. The extractions were performed at 100 °C, 3 h, 10 wt % solids loading, and 300 rpm.

Acid–base ratio characterization of the ILs employed in the screening is depicted in Table S17 in the SI. The ILs present acid–base ratios close to 1.00:1.00, from which most present acid–base ratios slightly lower than 1, from 0.95 to 0.99, except [Ch][MeSO_3_] with 1.07:1.00. Some interesting trends related to the relationship between the pH of the ILs and protein removal can be found by inspecting Figure 2. From the three acidic ILs—[MEA][HSO_4_], [Ch][HSO_4_], and [Ch][MeSO_3_]—only the [HSO_4_] ILs were able to remove the protein from the biomass. The explanation relies on the amount of acidic protons available in the medium. Acidic ILs with [HSO_4_]^−^ anions present a large pool of protons available for exchange. In a study by Firth et al.,^31^ they showed through density function theory (DFT) calculations that spontaneous proton dissociation from [HSO_4_]^−^ to water occurred for ammonium-based protic ILs, preceded by the formation of an anion trimer structure. However, with [Ch][MeSO_3_], the slight excess of methanesulfonic acid did not provide sufficient acidity to the system. Although the proteins were removed from the squid pen, the protein yield was near zero because the proteins most likely were hydrolyzed in the acidic media. The neutral IL, [MEA][MeSO_3_], was also inefficient in promoting protein solubilization. The alkaline ILs [Ch][OAc] and [MEA][OAc] were able to extract a considerable amount of protein. However, upon antisolvent addition to the protein–[Ch][OAc] mixture, instantaneous protein precipitation occurred (Video S1 in the SI), which confirms the superiority of this IL toward protein extraction and recovery. Of course, the pH trend does not solely explain the protein solubility and precipitation; other phenomena related to ion hydrophilicity, intermolecular interactions, and effect of cosolvent which will ultimately impact the salting-in and out effects of the IL will be covered in Section 3.8 with the MD simulations. Regarding the controls, NaHSO_4_ and NaOAc did not extract any protein (data not shown), which shows the pH and anion structure are not the main causes for protein extraction; the form in which these ions are (in solution or in an IL) also matter. Sodium hydroxide extracted a high amount of protein, however, the protein recovery was not as considerable (about 30%). The protein is most likely hydrolyzed under such alkaline conditions which may play a hint at the lower stability of protein in aqueous media compared to ILs.

### 3.3 Optimization of Protein Extraction for [Ch][OAc]

Once [Ch][OAc] was chosen, optimization of the protein extraction parameters was performed. Extraction time, solids loading, and temperature were chosen as optimization variables, and two main response variables were assessed: protein removal and protein yield. Protein extraction experiments performed under 100 °C did not yield any measurable amount of recovered protein. It can be argued that high temperatures are necessary to disrupt both the hydrogen bonding and covalent bonds between protein and chitin. In fact, past studies employed harsh conditions with 1 M NaOH under high temperatures (<80 °C) at prolonged times to fully remove proteins from squid pens.^11,12^ Sodium hydroxide is a much stronger alkali than [Ch][OAc] due to the presence of hydroxide anions in solution. Therefore, protein–chitin bond disruption can occur from 80 °C.

A new 2^2^ design (with only time and solids loading as parameters) was then extracted from the previous 2^3^ DoE, with three extra center point triplicates being added, 10 wt % solids loading, and 3 h extraction time. The DoE analysis (SI) showed that all parameters are significant, including time and temperature interaction. The statistical significance of the model term coefficients, analysis of variance (ANOVA), and Pareto chart for both protein recovery and removal is shown in the SI. The analysis showed that all of the model terms are statistically significant (*p* < 0.05). The first-order model fits the experimental data and predicts the response using the following equation:
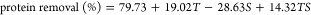
1
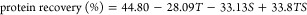
2where *T* and *S* correspond to the time and solids loading factors (dummy coded, i.e., from −1 to +1), and the *R*^2^ values for the protein removal and recovery were 0.99 and 0.70, respectively, indicating that the efficiency of protein removal is better predicted than how much protein is precipitated. Logically, protein removal is a simpler outcome variable, as it depends on only the mass of the samples before and after protein extraction. On the other hand, protein recovery is more complex because it involves both protein removal and their salting out via antisolvent addition. The surface response plots are shown in Figure S5 (SI). Protein removal was favored by long extraction times and low solids loadings.

Alternatively, protein recovery was favored by low solids and low extraction times. Long extraction times promote protein breakdown into oligopeptides, peptides, and amino acids, which may have remained in the IL even upon antisolvent addition. A compromise between high protein removal and high protein recovery must be achieved. A time course experiment (Figure S9, SI) showed 2 h of extraction was sufficient to ensure high protein yields (>60%). Therefore, an extraction time of 2 h and 5 wt % solids loading was chosen as the optimized condition.

The protein recovery as a function of the water content can be seen in Figure S12 (SI). A water content of lower than 5% was difficult to achieve by rotary evaporation due to the high hydrophilicity of [Ch][OAc], thus the initial water content was 10 wt %. Protein recovery remained high under a low water content and then decreased upon the addition of water to 60% protein recovery with a 40% water content. A higher water content promotes the salting out of the protein and therefore decreases the ionic liquid/protein interactions. As there were no significant differences between 10 and 20% water content, 20% was chosen as optimal for the IL recycling experiments. A higher water content in the IL decreases the cost related to solvent removal, once [Ch][OAc] can be synthesized via diluted aqueous solutions of either [Ch][OH] (46 wt %) or [Ch][HCO_3_] (80 wt %). An additional 2^2^ design with solids loading and water content was studied, and it showed that both parameters were significant and negatively affected the protein removal, therefore confirming that lower solids loading and water content are required to enhance protein removal and consequent chitin purity.

### 3.4 IL Recycling—Protein Build-Up

**Figure 3:**
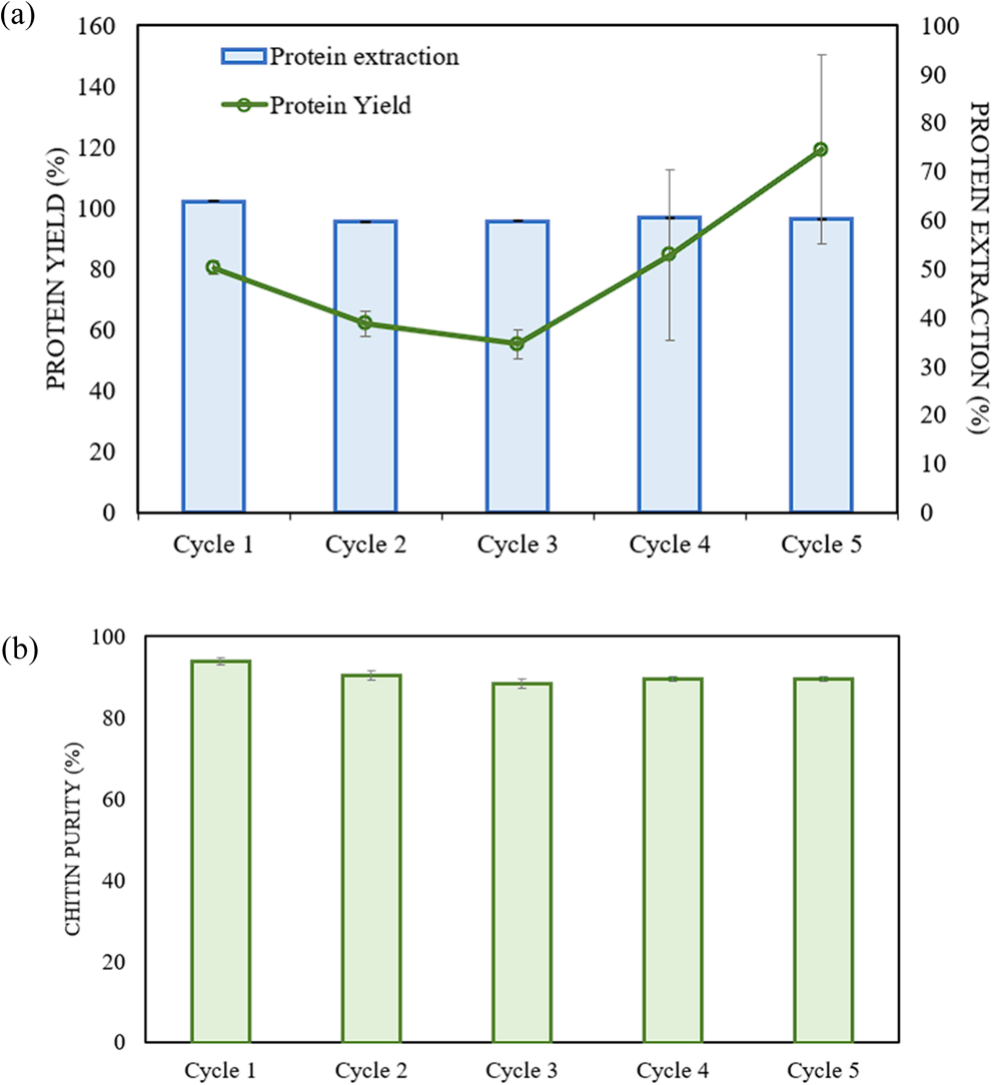
(a) Precipitated protein yield and extraction upon [Ch][OAc] recycling. The standard error was calculated based on triplicate analysis of the samples. (b) Chitin purity upon [Ch][OAc] recycling. The standard error was calculated based on triplicate of the samples.

Recycling of [Ch][OAc] was carried out for three samples along five cycles, and the protein recovery yields are reported in Figure 3. It is noticeable that the protein recovery drops after the first cycle to nearly 50% on the third cycle, showing a possible decrease in the efficiency. However, the yields sharply increase on the subsequent cycles up to 160%, which suggests that the proteins accumulate in [Ch][OAc] and after a certain threshold they precipitate back.

Although there has been a significant number of studies on IL or deep eutectic solvent (choline-chloride based) extraction of proteins,^32,33^ there is still a great gap in the investigation of their recovery and reuse, which poses difficulty to establish comparisons with other approaches. However, similar behavior can be observed in the lignin accumulation during the recycling of trialkyl ammonium hydrogen sulfate IL fractionation of lignocellulosic biomass, as observed by Brandt-Talbot et al.^34^ and Abouelela et al.^28^ Lignin can be salted out from the IL after fractionation upon the addition of excess water, using a method similar to that for squid proteins obtained in this work. The phenomenon underlying the lignin precipitation can be attributed to lignin cross-condensation along multiple pretreatment cycles^34^ which does not reflect what happened to the protein. However, the addition of water triggers changes in both ionic strength and pH of the medium, two parameters known to impact the solubility of proteins and facilitate the precipitation of amorphous protein aggregates.^35^ Lower ionic strength in aqueous choline-based IL systems is known to increase the likelihood of protein aggregation.^36^ A continuous process would yield a different protein accumulation pattern in the IL with a steady state being reached, minimizing the likelihood of sudden spikes in protein precipitation.

The IL recovery is also shown in Table S15 (SI). On average, most of the [Ch][OAc] was recovered with the washing steps of both chitin and protein. The slight losses along the recovery cycles are most likely due to the protein-bound IL via hydrogen bonding and electrostatic interactions. Wahlström et al.^37^ have shown that residual [Ch][Cl], up to 2.9 wt %, could be still found on the protein concentrate after dialysis on the extraction of Brewer’s spent grain protein with the deep eutectic solvent [Ch][Cl]/urea. Polesca et al.^23^ employed [Ch][OAc] to extract keratin from chicken feathers and managed to recycle the IL for four cycles without major losses in keratin extraction and IL recovery yields above 90 wt %. Nevertheless, they did not observe keratin accumulation on the IL along the cycles, which may be related to differences in protein polarity. Once keratin is known to be hydrophobic and more insoluble in solvents due to the high amount of disulfide bonds, salting out with ethanol/water mixtures promoted their full precipitation from the bulk of the IL.^38^ The [Ch][OAc] samples along the cycles have shown no signs of contamination (Figure S13 from SI) on their ^1^H NMR spectra, which shows that no major byproducts were produced upon the extraction.

### 3.5 Mass Balance for the Optimized Protein Extraction with [Ch][OAc]

**Figure 4:**
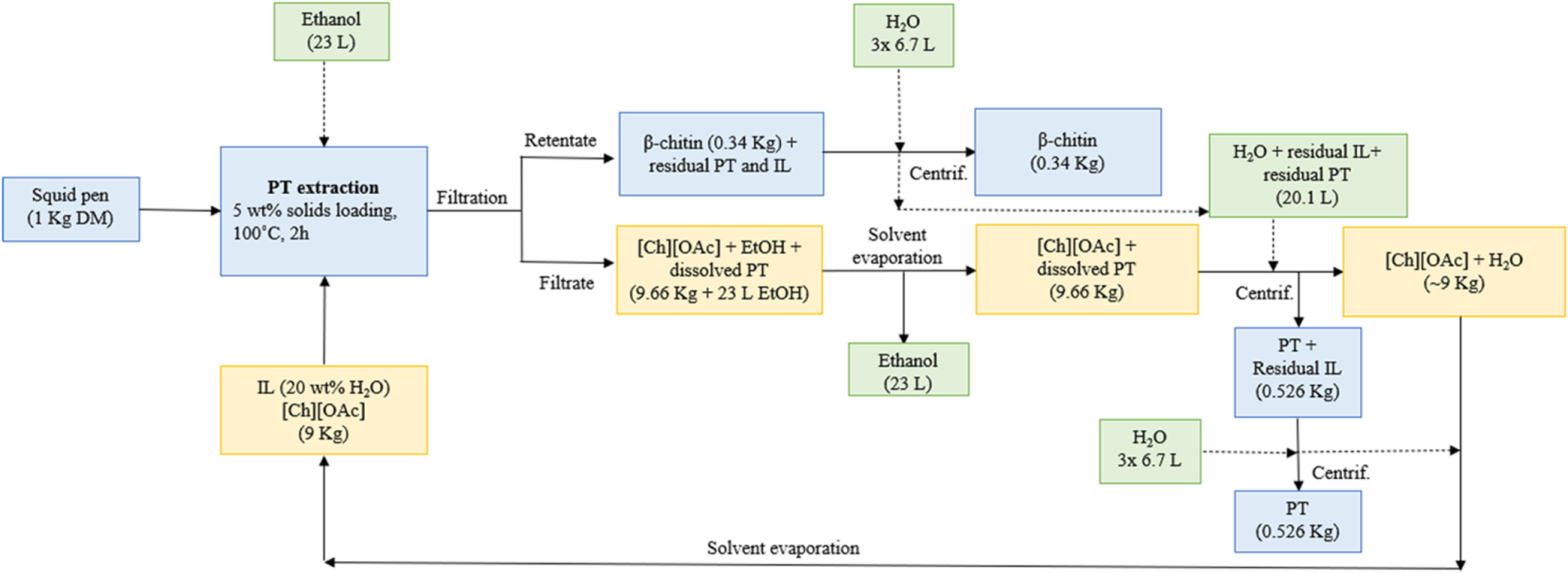
Mass balance for the optimized extraction of the squid pen protein (PT) with [Ch][OAc]. The blue boxes represent the feedstock and fractions streams. The yellow boxes represent the IL streams (only accounting for the IL dry weight) and the green boxes represent the cosolvent streams. The dashed lines represent the flow of cosolvents.

When developing a new process, it is essential to track mass flows for a better understanding of critical quantities such as biomass fraction yields and solvent usage. The mass balance for the optimized squid pen extraction with [Ch][OAc] is depicted in Figure 4. The β-chitin yield was 0.34 kg·kg^–1^ of squid pen, which is consistent with values obtained by Chaussard and Domard—between 30 and 35 wt %—on the conventional extraction of squid protein from squid pens.^39^ Cuong et al.^40^ also managed to obtain 35.8 wt % of chitin from *Loligo chenisis* pens via conventional alkaline extraction. McReynolds et al.^41^ and Lv et al.^42^ obtained nearly 30 wt % of β-chitin by using the alkaline deep eutectic solvent K_2_CO_3_/glycerol. Sulthan et al.^43^ employed the deep eutectic solvent ChCl/malonic acid to obtain nearly 43 wt % of β-chitin. Approximately, 0.53 kg of protein can be produced per kg of dry squid pen, which is a valuable yet multifunctional stream, due to its several amino acid constituents, and it can be obtained in a solid form, facilitating its preservation and storage. In terms of solvent consumption, nearly 40 L of water and 23 L of ethanol were employed per kg of dry squid pens, which is still a considerable amount. However, all of the solvent streams can be recycled, which does not generate wastewater.

Based on the flow rates presented in Figure 4, some green metrics factors have been calculated, such as *E*-factor, wastewater intensity (WWI), and solvent intensity (SI), as they represent well the amount of waste, wastewater, and solvent waste per kg of product. Although the process could be a closed loop with zero waste generation, these factors have been calculated considering a 97% solvent recovery to not underestimate the environmental impact of the process. Thus, an *E*-factor of 2.49, WWI-factor of 1.39, and SI-factor of 0.94 were estimated. Although the absence of comparable data from a similar process makes it challenging to interpret these values comprehensively, it is evident that they are all near zero. This suggests an optimal process with minimal to no waste generation. By comparing the calculated *E*-factor to those typically reported for different industrial sectors, a value of 2.49 is in the usual range calculated for bulk chemicals industries (1–5),^44^ agreeing with the obtained products in our proposed process from squid pen waste fractionation. It is also important to note that these reported values could be lowered by studying the scaling up of the process, as well as by incorporating process intensification considerations in future studies.

### 3.6 Materials Characterization Summary—β-Chitin and Protein

The extracted β-chitin presents a high degree of acetylation, as shown by ^1^H NMR estimation, 94%. This suggests that, despite the use of an alkaline IL, there was insufficient basicity to promote deacetylation of the *N*-acetyl groups. FT-IR analysis showed the main differences across the samples were related to the relative intensity of the bands. It was noted that the protein and the squid pen (which is mostly composed of proteins) present strong absorptions at 1630 and 1511 cm^–1^ due to the amide bands from the peptide bonds. However, with β-chitin, even though present, chitin contains *N*-acetyl groups, which are amides, such bands are lower in intensity. TGA revealed a trend in which, similarly to the FT-IR spectra, the squid pen had intermediary behavior between the two components (chitin and protein) where *T*_onset_ for the squid pen was 318 °C, for the β-chitin, 326 °C, and for the squid protein, 276 °C. Additionally, the TGA pattern for the squid pens resembled more protein since it is the primary component. The XRD analysis revealed that the CrI of the β-chitin from [Ch][OAc] extraction was higher (82%) than that measured from the conventional extraction method with NaOH (50–70%). The electrophoretic profile of some of the squid proteins obtained in this study showed that the proteins presented similar electrophoretic patterns with two distinct bands at 10 kDa and between 15 and 25 kDa. Such bands are likely to be related to muscular proteins from squid and which are typically present at low molecular weight.^45^ These values place the [Ch][OAc] fractionation as one of the best methods to produce high-purity streams from squid waste. The squid protein concentrate obtained in this work had an overall protein content (based on the amino acid profiling analysis) of 80%, excluding tryptophan. The total protein concentration from the amino acid profiling is lower due to the reduction in protein content from the two-step oxidation and acid hydrolysis. The primary essential amino acids detected were histidine, leucine, and valine, whereas tyrosine, proline, and alanine made up the majority of nonessential amino acids. Based on the amino acid profile (Table S16 from SI), the nitrogen-to-protein conversion factor was 5.95. The elemental analysis of the protein showed a nitrogen content of 14.4 ± 0.1%, which corresponds to a protein purity of 85.7 ± 0.6%, quite close to the 80% obtained from the amino acid profiling.

**Figure 5:**
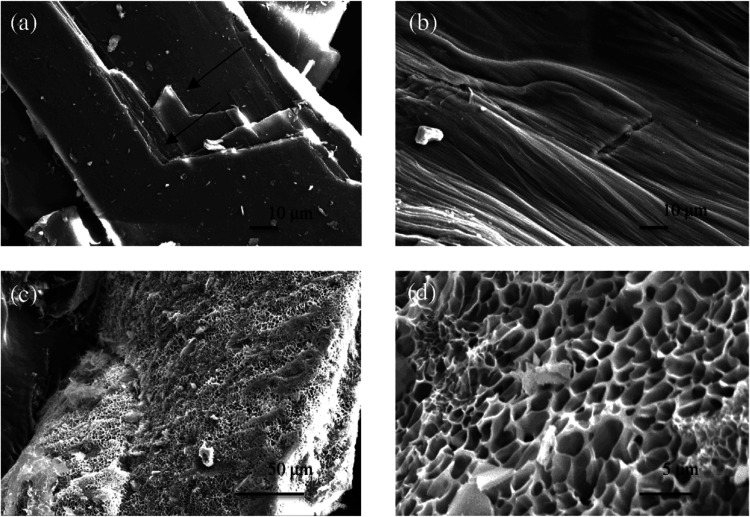
SEM surface image of (a) the squid pen; (b) β-chitin after protein extraction with [Ch][OAc]; (c) squid pen protein under low magnification; (d) squid pen protein under high magnification.

The squid pen surface is slightly rough and is present in layers, as visible in Figure 5a. These layers form as a result of the squid’s secretion during their formation.^11^ Removal of these superficial layers through treatment with [Ch][OAc] reveals a smooth and groovy surface on the β-chitin, as observed in previous studies.^11,46^ The freeze-dried squid pen protein is highly porous with nanosized pores distributed across its surface (Figure 5c,d). These pores were most likely formed during the freeze-drying process and resemble the formation of hydrogels made of animal protein^47^ and soy protein.^48^

### 3.7 Applications for the Fractions

#### 3.7.1 Squid Pen Protein

As a simple proof of concept for the squid protein utilization as a biomaterial for potential packaging applications, biocomposite films with α- or β-chitosan and squid pen protein (2:1:0.3 chitosan/protein/glycerol mass ratio) were produced by solution casting in 3 wt % acetic acid (Figure S2 in the SI). The biocomposite films were not very thin (average 140 μm for both types) and were quite strong with an average of 93.15 ± 7.9 and 83.5 ± 6.2 MPa of tensile strength at break for the α- and β-chitosan, such values were superior to the ones obtained by Cuevas-Acuña et al.,^49^ 58.1 ± 5.3 MPa, with an α-chitosan/squid skin gelatin ratio of 90:10 or by Ferreira et al.,^50^ 50 MPa, with 14 wt % whey protein and 86% α-chitosan. However, pure α-chitosan films such as the ones obtained by Xu et al.^51^ are still mechanically stronger—ranging from 116 to 118 MPa—than the blends from this work. The contact angle of the films showed they were hydrophilic with 78.8 ± 0.44 and 64.7 ± 3.33° for the α- and β-chitosan, whereas pure α-chitosan films present slightly more hydrophobic features such as the values obtained by Xu et al.^51^ that vary from 86.9 to 91.7°. Ideally, enhanced hydrophobicity is desired in films to avoid the permeation of water into the package. Therefore, the film formulation needs to be further explored in the search for more hydrophobic materials.

Amino acid profiling (Table S16 in the SI) of the protein isolate showed that the major essential amino acids were histidine, leucine, and valine, which show that the protein can be a source of valuable amino acids for aqua culture^52,53^ and applications in human feed is also a potential avenue.^45^

#### 3.7.2 β-Chitin

Although it can be directed toward β-chitosan production—which would add an additional environmental burden due to its high waste generation^54^—β-chitin can be electrospun into scaffolds or mats for biomedical applications such as wound dressings, drug delivery agents, or bone-growth scaffolds.^55^ For instance, Gomes et al.^56^ employed the same IL used in this study, [Ch][OAc], to produce biocompatible, highly porous, and interconnected sponges from crab shells α-chitin. Biocompatibility was probed by assessing the metabolic activity of L929 fibroblasts and seeding human adipose stem cells on the sponge surface. Despite a solvent-intensive synthesis, chitin nanowhiskers have been employed as reinforcing agents in elastomers synthesis, either in epoxidized natural rubber,^57^ in elastomeric composites with polycaprolactone^58^ or modified polyurethanes.^59^

### 3.8 Molecular Dynamics (MD)

The phenomena behind the squid protein extraction with ILs at the molecular level can be quite complex due to the variety of inter- and intramolecular interactions involved. Nevertheless, past studies on the interaction of proteins with ILs have shown cooperative and competitive effects of IL solvation of proteins can be rationalized.^35,60,61^ Squid proteins, especially muscular ones such as actin, myosin, and tropomyosin, are known to thermally denature from 40 to 80 °C.^11,62,63^ IL treatment at 100 °C most likely caused the squid pen proteins to denature. Bui-Le et al.^61^ have also shown that proteins such as the green fluorescent protein are more easily denatured in the presence of pyrrolidinium-based ILs, which may reinforce that protein denaturation may have happened even below 100 °C, which then caused their unfolding and exposure of the hydrophobic core. The acetate anion presents a high value of the Kamlet–Taft parameter β when compared with [MeSO_3_] and [HSO_4_],^64^ which is related to the hydrogen bond basicity; it is known that high β values may lead to protein destabilization.^35^ Once denatured, the protein remains soluble in the IL and upon the addition of ethanol as a cosolvent. After ethanol evaporation and the addition of excess water, the protein was salted out from the IL due to the stronger IL–water interactions. The goal of these MD simulations was 2-fold: first, to understand why protein solubilization was more effective in ionic liquids with the [OAc] anion. Second, we need to rationalize why the combination of [OAc] with the [Ch] cation allows optimal recovery of the proteins.

#### 3.8.1 Anion–Protein Interactions

**Figure 6:**
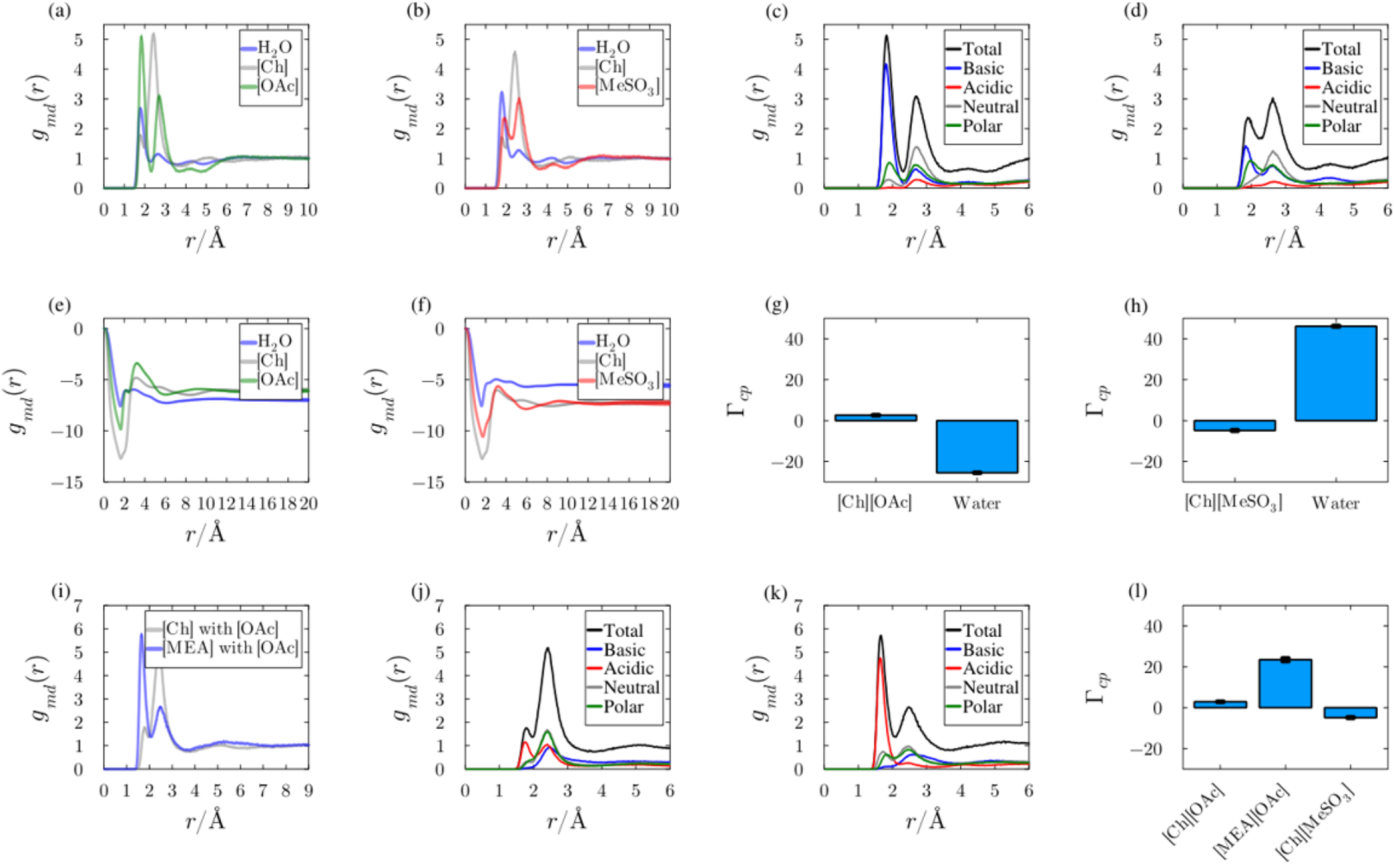
Protein solvation in IL systems using minimum distance distribution functions (MDDFs) and the Kirkwood–Buff (KB) theory of solvation. The figures enclosed in the black box show the distributions and KB integrals of the anions [OAc]^−^ and [MeSO_3_]^−^ in the presence of the same cation. The figures enclosed in the blue box refer to the distributions of the cations [Ch]^+^ and [MEA]^+^ in the presence of a shared anion. (a, b) MDDFs for [Ch] and [OAc] and [Ch] and [MeSO_3_], respectively. (c, d) Stronger association with basic residues, particularly for [OAc]^−^. (e, f) KB integrals for both IL systems, and (g, h) comparison of the preferential solvation parameters. (i–k) Comparative analysis of protein solvation using the [OAc]^−^ anion with [Ch]^+^ and [MEA]^+^ cations. (l) Contrasting the preferential solvation parameters between ILs with different cations, underscoring the variance in solvation preference and interaction strengths within these complex systems.

Minimum distance distribution functions (MDDFs) for two distinct IL systems sharing the same cation are listed in Figure 6. The MDDFs peaks indicate the regions with the highest probability of locating the compound of interest in the solution relative to the protein surface.^65,66^ The MDDFs for the anions exhibit significant differences in the systems. Specifically, in the [OAc]^−^ MDDF (Figure 6a), a sharp peak appears at ∼1.9 Å, showing a high probability of acetate–protein hydrogen bonds.^60,67^ Another important [OAc]^−^ MDDF peak appears at ∼2.9 Å and is a result of van der Waals interactions with the protein or interactions mediated by other ions or water molecules. The position of the cation peak, which intermediates the two anion peaks, suggests alternating charge solvation layers and thus a strong cation–anion cooperative solvation effect. The accumulation of [MeSO_3_] in [Ch][MeSO_3_] (Figure 6b) is less pronounced than that of [OAc], being the second peak greater than the first, and both smaller than that of the cation. Clearly, [OAc] has a stronger binding to the protein surface than [MeSO_3_].

Protein residue-type contributions for the total MDDF distribution of ions are displayed in Figure 6c,d. In Figure 6a, the first peak of the MDDF of [OAc]^−^ results from interactions with basic protein residues, mediated by interactions with acetate oxygen (Figure S5). In contrast, [MeSO_3_]^−^ exhibits a relative probability of interactions with basic residues smaller than that of [OAc]^−^. One can note that in Figure 6d the MDDF shows significant interactions with basic residues at hydrogen bonding distances; however, such contribution to the total MDDF is also smaller than specific interactions displayed by the [OAc]^−^ anion.

Kirkwood–Buff integrals (KBIs) for the [Ch][OAc] and [Ch][MeSO_3_] IL systems are shown in Figure 6. In brief, when the water KBI exceeds that of the ions, it indicates a greater relative affinity of water for the protein surface. In the case of acetate, the ions’ KBIs exhibit a stronger affinity for the protein surface, whereas for [MeSO_3_]^−^, the opposite trend is observed. This means that the IL with [OAc]^−^ preferentially solvates the protein relative to water, while the protein is preferentially hydrated when the anion is [MeSO_3_]^−^, as shown in Figure 6c,d. The greater preferential solvation parameter implies that a greater number of IL ions are found around the protein for the system with [Ch][OAc] than with [Ch][MeSO_3_], for the same bulk concentration. In general, solutes that dehydrate proteins induce protein denaturation and may facilitate protein solubilization, as the protein tends to expose its surface to the solution.

#### 3.8.2 Cation–Protein Interactions

The distributions of components in solutions with different cations but sharing the same anion are shown in Figure 6i–k. The shared anion is [OAc]^−^ and either [MEA]^+^ or [Ch]^+^ is the cation. The distributions of [Ch]^+^ (in gray) and [MEA]^+^ (in blue) display two primary peaks at approximately 1.8 Å, resulting in the formation of hydrogen bonds with the protein’s surface. While both cations exhibit MDDFs with two peaks within 5 Å of the protein surface, the first peak is notably more pronounced for [MEA]^+^. This suggests a higher likelihood of [MEA]^+^ forming hydrogen bond interactions with the protein, particularly with acidic residues (Figure 6k). This significant contribution from acidic residues is not observed when the cation is [Ch]^+^, as shown in Figure 6j. The inability of [MEA]^+^ to induce protein precipitation, despite its solubilization capability, might be inferred from Figure 6l: Both ILs with these cations preferentially solvate the protein and thus interact favorably with the protein surface relative to water. Yet, the preferential solvation by [MEA]^+^ is significantly stronger than for [Ch]^+^. This potentially explains the difficulty in precipitating the protein from the solutions with [MEA]^+^. While the IL containing [Ch]^+^ also preferentially solvates proteins compared to water, its comparative affinity to the protein surface is much weaker than that of [MEA]^+^, thereby facilitating protein recovery.

### 3.9 Technoeconomic Analysis

Technoeconomic analysis was conducted to evaluate the effects of the protein extraction process. These assessments are particularly crucial to minimizing solvent consumption for environmental and economic issues, as ethanol and water are introduced at various stages of the process to facilitate the recovery of chitin and protein. Consequently, energy is required for the evaporation of these substances to achieve the appropriate composition for the recovery of the IL for reuse. The IL recovery was set to 97%, following previous results shown in Table S15 (SI). In addition, the experimental values reported in Figure 4 were considered for the estimation of heat consumption for ethanol and water evaporation.

The simulation takes into account heating the squid pen and IL mixture to 100 °C for protein extraction. Then, ethanol evaporation was simulated by a flash vessel at 0.1 bar, optimized by trial and error, until no ethanol was detected in the bottom stream (IL + water). No IL was recovered in the top stream. 1.1 kg water·kg^–1^ of IL as low-pressure steam was necessary to provide the heat needed for the distillation. The top stream (ethanol) was condensed and then cooled to room temperature and recirculated, recovering the excess heat for its employment in subsequent separations. Two-step multiple-effect evaporators were used for water removal and IL recovery. Pressure in both vessels (0.3 and 0.1 bar in the first and second vessels, respectively) was optimized by trial and error to minimize vacuum and energy consumption and ensure that the recovered IL matched the required characteristics for its reuse. The excess heat from ethanol condensation was used in the first step, as well as an additional 1.78 kg water·kg^–1^ of IL as low-pressure steam for heating needs. Excess heat from water condensation at the top of the vessel was recovered and employed in the second step with no additional heating needs. Water was cooled to room temperature and recirculated. IL was then heated up again to 100 °C and recirculated. From the simulation, annual costs were calculated as described in the methodology section. The cost breakdown showed that the heat needed for IL recovery dominates at 64% of the total costs, followed by fixed costs at 17%. Smaller contributions come from feedstock (7%), IL makeup (6%), and other factors such as annualized CAPEX (2%), heat for the reactor during extraction (3%), and cooling water (1%). Electricity accounted for only 0.2%.

As expected, the most significant contribution is the heat needed for solvent recovery due to the high volumes of ethanol and, mainly, water (85 wt % after mixing with the IL) employed in the process. This is especially important considering that the heat of vaporization of water is 2.65 times higher than that for ethanol. For this, energy consumption in the multiple-effect evaporators for water evaporation is 1.6 times higher than that for ethanol removal, accounting for 40% of the minimum protein selling price. Therefore, new methods for IL recovery are worth investigating to improve the energy efficiency of the process. IL makeup costs were calculated considering a 97% IL recovery from experimental results in laboratory, which may not completely translate to a larger scale.^68^ The large volumes of solvents employed for relatively low productivity lead to high total field costs (due to equipment), which impact the fixed costs, significantly contributing to the minimum selling price. The main contributors to these costs are the three evaporators employed in ethanol and water distillations, accounting for 15% of all fixed costs. Finally, it is important to note that feedstock costs are estimated using a squid pen powder price that may not be current, given the challenge of accessing updated pricing information. Hence, caution is advised in considering this aspect, as there is potential for cost reduction if the plant is integrated into the fish industry, where squid pens are commonly viewed as waste material.

According to our simulation and considering all of the costs explained in the methodology section, the minimum protein selling price obtained is $9 kg^–1^, which was calculated considering a protein productivity of 416 ton·year^–1^, in accordance with the mass balance (Figure 4) for simulated flow rates. However, it is worth mentioning that this price was calculated considering only protein and does not take into account the possible income from selling β-chitin as a byproduct. Thus, based on chitin productivity and considering a price of $3.75 kg^–1^,^69^ the protein selling price could be lowered to approximately $6.6 kg^–1^. In terms of environmental impact, the process generates CO_2_ emissions of 4.27 kg of CO_2_ per kg of products, factoring in the production of both protein and β-chitin, which account for 61 and 39% of CO_2_ emissions, respectively. The predominant source of these CO_2_ emissions is the significant energy requirements of the solvent recovery process, compounded by the limited availability of heat sources beyond steam. This underscores the critical need to explore alternative methods for recovering ILs from water streams given their substantial impact on both economic and environmental considerations. A more flexible method for IL dehydration is pervaporation, a scalable membrane separation technique that can be selective toward the direct recovery of volatile solutes or solvents from nonvolatile solvents in a quantitative manner.^70^

## 4 Conclusions and Perspectives

Designing ILs for protein extraction requires the appropriate selection of cation and anion combination. Squid pens are an excellent model for a shellfish biorefinery because they present a more simple composition when compared to crabs, prawns, and lobsters. In this work, an ionic-liquid-based process with [Ch][OAc] was designed and optimized that can generate two product streams of high purity, squid protein and β-chitin. Recycling of the IL showed that protein accumulates for a number of recycles and can eventually be recovered, without compromising the efficiency and purity of the IL. The mass balance of the process revealed that the solvent usage is high; however, process integration and IL recycling can ensure greener credentials for the process. Material characterization of the two streams showed that the β-chitin presents a low protein content and is highly crystalline, whereas the protein isolate has a high protein content and low molecular weight. MD simulations unveiled the reasoning behind the superiority of [Ch][OAc] to extract and precipitate protein from aqueous solutions in comparison to other ILs screened with the same cation or anion, underpinning the role of the anion on the protein extraction and the role of the cation on the protein precipitation. Technoeconomic analysis showed how energetic requirements for washing steps and IL recovery can heavily impact the protein minimum selling price. The coproduction of β-chitin decreases the minimum selling price of the protein. Solvent usage impacts negatively on CO_2_ emissions and emphasizes that despite its promise, an IL-based refinery requires further design process integration and optimized heat recovery, as well as increased efficiency in separation processes. This work can be viewed as the foundation of an IL-based biorefinery for shellfish, where future works on carbonate-rich shellfish such as crabs, prawns, and lobster can be beneficial for the holistic utilization of shellfish waste.

## Supporting Information Available

Feedstock and extracted chitin and squid pen protein; squid pen protein films; detailed description of the factorial design of experiments, analysis of ^1^H NMH, FT-IR, TGA, SEM, XRD, SDS-PAGE, CHN analysis, amino acid profiling, film thickness, tensile strength, and contact angle; calculations of yields and purity of extracted chitin and protein; statistical data analysis of the optimization experiments; and detailed description of the MD simulations and technoeconomic analysis (PDF)
Protein precipitation from the IL (Video S1) (MP4)
