# Supplementary material for: Fractionation of Squid Pens with Ionic Liquids—An Upgraded β-Chitin and Shellfish Protein Production
Source: ACS Sustain Chem Eng. 2025 Feb 12;13(7):2649–60. doi: 10.1021/acssuschemeng.4c04217 (PMC11863544; doi:10.1021/acssuschemeng.4c04217)
Supplement: Supplementary file 1 — sc4c04217_si_001.pdf [file sc4c04217_si_001.pdf]

Electronic Supplementary Information – ESI

Fractionation of Squid Pens with Ionic Liquids – An Upgraded β-Chitin and Shellfish Protein Production

Pedro, Y.S. Nakasu[1]; Vinicius Piccoli[3]; Antonio Ovejero-Pérez[1]; Priyanka Kumar[1]; Amir Al Ghatta[1]; Susiana Melanie[2]; Cariny Polesca[4]; Leandro Martinez[3]; Jason P. Hallett[1].

1. Department of Chemical Engineering, Imperial College London, SW7 2AZ, London- UK
2. Department of Materials, Imperial College London, SW7 2AZ, London- UK
3. Department of Chemistry, State University of Campinas, CEP 13083-862, Campinas-BR.
4. CICECO – Aveiro Institute of Materials, Department of Chemistry, University of Aveiro, 3810-193 Aveiro, Portugal

Number of pages: 35. **Number of Figures:** 19. **Number of Tables:** 17.

1. Materials and Methods

1.1 Feedstock, β-chitin and protein images

(a) (b) (c)

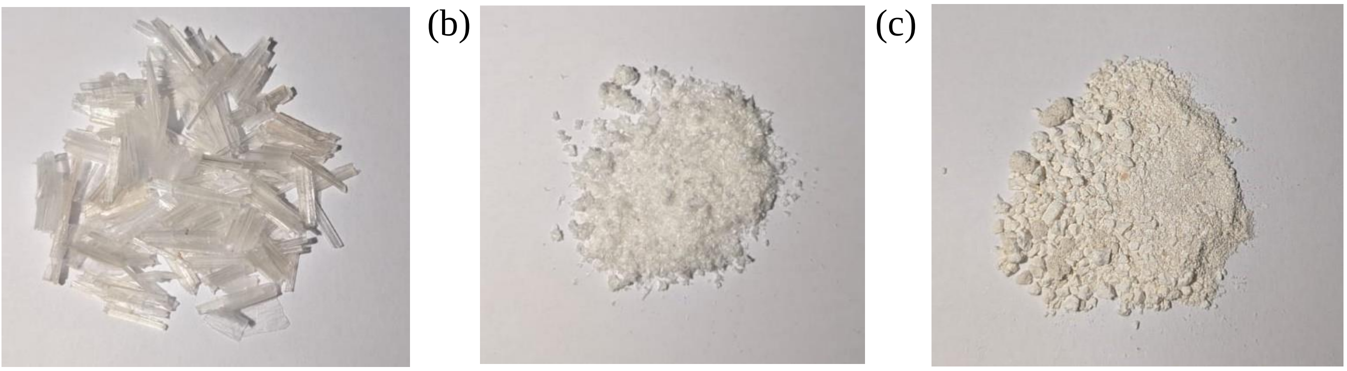

Figure S1. (a) Squid pen; (b) β-chitin powder after [Ch][OAc] extraction; (c) Squid pen protein after [Ch][OAc] extraction.

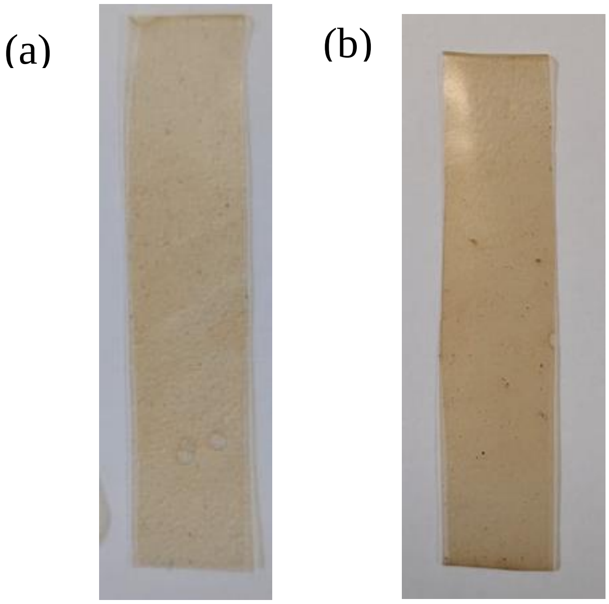

Figure S2. (a) α-chitosan and squid pen protein films (b) β-chitosan and squid pen protein films.

2. Video of protein precipitation

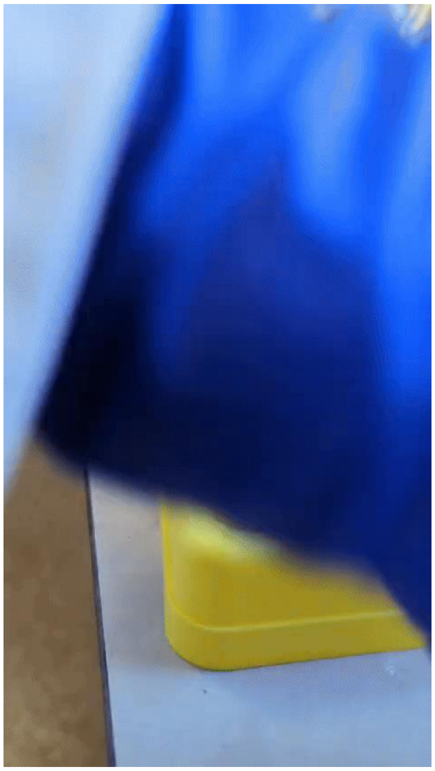

Video S1. Precipitation of squid protein from [Ch][OAc].

3.1 Optimization experiments

3.1.1 Factorial design

A 2^3+3 (center points) factorial design of experiments (DoE) was employed to understand the impact of temperature, extraction time, and solids loading on both protein yield and β-chitin purity. The parameter ranges (Table S1 from ESI) were: temperature – 25, 62.5, and 100 ˚C; extraction time – 1, 3, and 5 h; and solids loading – 5, 10, and 15 wt%. The 2^2 design (with time and solids loading as parameters) extracted from the previous 2^3 DoE had three extra centre point triplicates being added, 10 wt% solids loading and 3 h extraction time (Table S2 from ESI). The reaction setup was similar to section 2.3 with a fixed amount of 1 g squid pen (on a dry basis).

3.1.2 Single variable optimisation – Water content and extraction time

The impact of water content was studied in a single variable optimization experiment consisting of four water content runs in triplicates, 10, 20, 30, and 40 wt% at 100˚C, 10 wt% solids loading for 3h. Similarly, the extraction time was studied from 1, 2, 3 and 4 h in triplicates at 100˚C, 5 wt% solids loading, and 20 wt% water content.

4.2 Materials Characterization

4.2.1 Proton nuclear magnetic resonance - ^{1}H-NMR

Acid-base ratios of [$MeSO_3$], and [OAc]-based ILs were determined via ^{1}H-NMR on a ECZ 400S 400 MHz spectrometer (JEOL, Tokyo, Japan), according to Nakasu et al. (2020)[1]. The degree of acetylation of β-chitin was estimated based on ^{1}H-NMR with the same 400 MHz spectrometer after solubilization in 30 v/v% DCl/D_2O[2].

4.2.2 Fourier Transform Infrared Spectroscopy – FT-IR

FT-IR was recorded using a Cary 630 spectrometer (Agilent, California, USA) in the Attenuated Total Reflectance (ATR) mode.

4.2.3 Thermogravimetric Analysis – TGA

TGA of β-chitin and protein was performed to understand their thermal decomposition behavior using a TGA Q5000 V3.17 Build 265 (TA Instruments, Delaware, USA). Briefly, 3 mg of each sample was initially heated up to 100 ℃ and left to equilibrate for 30 seconds. Consequently, samples were heated at a heating rate of 20 $℃·min^{-1}$ up to 600 ℃ under nitrogen.

4.2.4 Scanning Electron Microscopy (SEM)

Scanning electron microscopy was performed in a JSM 6010LA microscope (JEOL, Tokyo, Japan) was used to examine the surface morphology of the freeze-dried β-chitin, protein and squid pen. The samples were gold-coated before being analyzed by SEM. Secondary electron imaging (SEI) was used with an accelerating voltage of 15kV.

4.2.5 X-ray powder diffractometry (XRD)

XRD analysis was carried out in a D2 Phaser diffractometer (Bruker, Massachusetts, USA) to analyse the crystallinity of the β-chitin and squid pen. The instrument was operated at 2θ 5-40 and with step size 0.018 and non-spinning mode with Cu Kα radiation with λ = 1.542 Å.

4.2.6 Sodium dodecyl-sulfate polyacrylamide gel electrophoresis (SDS-PAGE)

SDS-PAGE of the extracted squid protein was performed with an Invitrogen Mini Gel Tank (Thermo Fisher Scientific, UK). The polyacrylamide separation gel used was the Invitrogen Novex Tris-Glycine Mini Gel (16% polyacrylamide 1.0 mm thick with 15 wells, Thermo-Scientific). The protein was dissolved at varying concentrations (3, 5, 8, and 10 mg mL^{-1}) in a buffer solution of 0.05 M Tris-HCl (pH 8.6), 8 M urea, and 10 mM of the reducing agent dithiothreitol (DTT), then stirred for 2 h. The samples were then mixed with a dye buffer (20% glycerol, 4% SDS, 10 mM DTT, 0.5 M Tris-HCl pH 6.8, and 0.02% bromophenol blue) to make 40 uL final samples of protein solution and dye buffer in two different ratios, 5:2 and 1:1, before heating at 90°C for 5 min for complete protein denaturation. A molecular weight marker, PageRuler Low

Range Unstained Protein Ladder (Thermo Fisher Scientific, UK) and the samples were then loaded onto the gel and submitted to a rung at 80 V for 1 h, followed by 120 V for 1 h. Subsequently, the gel was stained using Coomassie Brilliant Blue G-250 overnight at room temperature in a procedure comparable to Polesca et al. (2023)[3].

4.2.7 CHN analysis

CHN content of the samples was determined via the Dumas method. The analysis was performed externally via Medac Ltd. (Woking, UK).

4.2.8 Amino acid profile

Amino acid profiling of the squid protein was carried out by Sciantec (Sciantec Analytical Services Limited, UK), excluding tryptophan. The customized Nitrogen Factor of both protein products was then calculated using their amino acid profiles, following the steps described in Hames et al. (2008)[4]. Brifely, the sample is oxidised with a combination of hydrogen peroxide, formic acid and phenol. This converts any methionine to methionine sulphone and any cystine to cysteic acid to avoid losses upon hydrolysis. Excess oxidation reagent is decomposed with sodium metabisulfite. The oxidised sample is hydrolysed with hydrochloric acid and the amino acids are then separated by ion exchange chromatography and determined by post column reaction with ninhydrin using photometric detection.

4.2.9 Film thickness

Thickness measurements were carried out with a digital caliper (Whitworth 0-150mm METR-ISO, UK) at three random positions on each film. Only films with smooth surface and thickness showing a standard deviation of less than 10% were selected for further mechanical testing. The values of these sections were used for mechanical analysis.

4.2.10 Tensile Strength

Each film specimen (5 cm × 1 cm) was fixed between the separated grips (20 mm) of the universal material testing machine (EZ50 Lloyd instruments/Ametek, U.S.) with the crosshead speed set to 0.1 mm/s. Tensile strength was calculated as the maximum load divided by the initial cross-sectional area of the sample and expressed in MPa. Elongation at break was calculated as the ratio of the elongation at the point of the sample rupture and the initial length of samples multiplied by 100. The measurements were performed in triplicate.

4.2.11 Contact angle

Contact angle was measured using a drop shape analyser (DSA30S, Krüss, Germany) and ADVANCE software (Krüss, Germany). A 2 μL sessile drop was applied to the film surface and allowed to stabilise, for 1-3 seconds. Once stable, a 30 second measurement was taken of each film, and an image was captured at the 1, 15 and 30 second mark using a camera (UI-3060CP Rev. 2, IDS, UK).

4.3 Statistical Analysis

All data collected from the optimization experiments was subjected to statistical tests. ANOVA was performed on the factorial designs as well as linear regressions. The statistical tests were applied with the software STATISTICA 10.0 (Statsoft, Oklahoma, USA).

5. Calculations

5.1 Protein removal

Protein removal was calculated based on gravimetry:

Protein removal (%) $=(1 - Residual\ PT\ in\ chitin) \cdot 100\%$

$$= \left(1 - \left(\frac{m_{Chitin\ residue} - m_{chitin\ sample}}{m_{sample} \cdot PT_{\%}}\right)\right) \cdot 100\% \qquad (1)$$

Where m_{Chitin} residue is the mass of the chitin residue after protein extraction, $m_{chitin\ sample}$ is the mass of chitin in the original sample, m_{sample} is the mass of squid pen in dry basis, and PT% is the protein content in the squid pens.

5.2 Protein recovery

Protein recovery was calculated based on gravimetry:

$$\text{Protein recovery} = \left(\frac{m_{PT}}{m_{PT\ in\ sample}}\right) = \left(\frac{m_{PT}}{m_{sample} \cdot PT_{\%}}\right) \qquad (2)$$

Where m_{PT} is the mass of recovered protein, m_{sample} is the mass of squid pen in dry basis, and PT% is the protein content in the squid pens.

5.3 Nitrogen to protein conversion factor

The estimation of this parameter was performed according to Gosukonda et al. (2020) [5], where the factor was calculated as a weighted average of the nitrogen content from each of the amino acids present in the amino acid profile.

5.4 Purity estimation of protein samples

The purity of the protein samples was estimated via CHN analysis. A nitrogen to protein conversion factor of 6.25 has been used based on previous studies on giant squid [6] and cuttle fish [7].

$$PT_{Purity} = N_{PT} \cdot 5.95\ \% \qquad (3)$$

Where N_{PT} is the nitrogen content of the protein sample.

5.5 Purity estimation of chitin samples

The purity of the chitin samples was estimated via gravimetry:

$$Chitin_{Purity}(\%) = \left(1 - \left(\frac{m_{chitin} - 0.30 \cdot m_{squid\ pen}}{0.686 \cdot m_{squid\ pen}}\right)\right) \times 100\% \qquad (4)$$

Where m_{chitin} is the dry mass of β-chitin obtained after extraction, 0.30 corresponds to the β-chitin mass fraction content after alkaline treatment (1M NaOH at 80°C for 3h) in the squid pen, 0.686 corresponds to the mass fraction content after alkaline treatment (1M NaOH at 80°C for 3h) in the squid pen and $m_{squid\ pen}$ is the dry mass of squid pen before extraction.

5.6 Crystallinity index, CrI

The CrI was determined using the following equation according with Berger et al. (2014)[8] :

$$CrI\ (\%) = 100 \times \left\{\frac{(I(\theta_c)-I(\theta_a)))}{I(\theta_c)}\right\} \qquad (5)$$

Where $I(\theta_c)$ is the relative intensity of the crystalline regions (2θ = 20°) and $I(\theta_a)$ corresponds to amorphous regions (2θ = 12°) for the samples.

6. Statistical analysis

6.1 Design table

Table S1. First 2^3 factorial design with 3 center points triplicates.

**Time (h)**	**T (˚C)**	**Solids loading (wt%)**
5	100	15
5	100	5
5	25	15
5	25	5
1	100	15
1	100	5
1	25	15
1	25	5
3	62.5	10
3	62.5	10
3	62.5	10

Table S2. Second 2^2 factorial design with new center point triplicates.

**Time (h)**	**Solids loading (wt%)**
5	15
5	5

1	15
1	5
3	10
3	10
3	10

6.2 Summary of effects for the 2^2 factorial design with three center point triplicates – Factors: Time and solids loading

6.2.1 Effect Estimates for Protein Removal:

Table S3. Effect estimates, p value and confidence intervals. R^2 = 0.995; Adjusted R^2: 0.991

	Effect	Std. error	T(3)	p	-95% Conf. limit	95% Conf. limit	Coeff	Std. error Coeff
**Mean/Intercept**	79.73	0.41	195.90	0.000026	77.98	81.48	79.73	0.53
**(T) Time**	19.02	1.08	17.67	0.0032	14.39	23.66	9.51	0.71
**(S) S. Loading**	-28.63	1.08	-26.59	0.0014	-33.27	-24.00	-14.32	0.71
**(1) by (2)**	14.32	1.08	13.30	0.0056	9.69	18.96	7.162	0.71

6.2.2 ANOVA table for Protein Removal:

Table S4. ANOVA for the effect of time and solids loading on Protein Removal.

	SS	df	MS	F value	p value
**(1) Time**	361.95	1	361.95	312.13	0.0032
**(2) S. Loading**	819.96	1	819.96	707.11	0.0014
**(1) by (2)**	205.21	1	205.20	176.96	0.0056
**Lack of fit**	3.69	1	3.69	3.18	0.22
**Pure error**	2.31	2	1.16		
**Total SS**	1393.13	6			

6.2.3 Pareto Chart for Protein Removal:

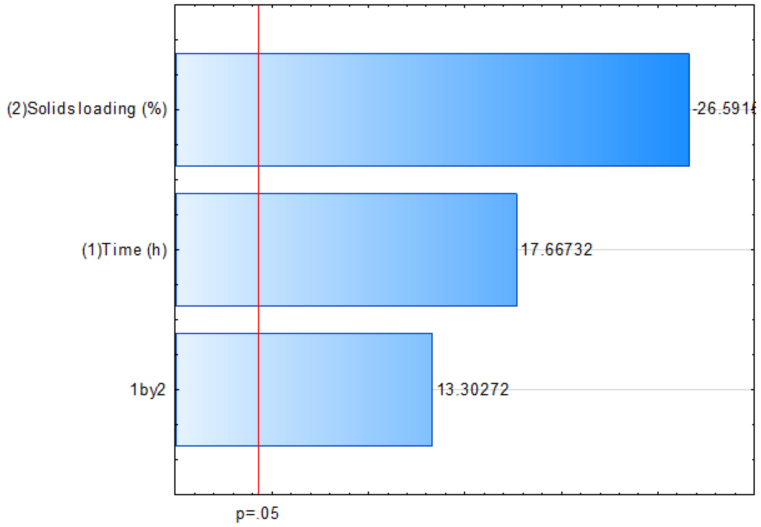

Figure S3. Pareto chart for the effect of time and solids loading on protein removal.

6.2.4 Effect Estimates for Protein Recovery:

Table S5. Effect estimates, p value and confidence intervals. $R^2 = 0.696$; Adjusted R^2: 0.392

	Effect	Std. error	T(3)	p	-95% Conf. limit	95% Conf. limit	Coeff	Std. error Coeff
**Mean/Intercept**	44.80	2.40	18.68	0.0028	34.48	55.12	44.80	2.40
**(1) Time**	-28.09	6.35	-4.43	0.047	-55.39	-0.78	-14.04	3.17
**(2) S. loading**	-33.13	6.35	-5.22	0.035	-60.43	-5.82	-16.56	3.17
**(1) by (2)**	33.8	6.35	5.33	0.033	6.49	61.10	16.9	3.17

6.2.5 ANOVA table for Protein Recovery:

Table S6. ANOVA for the effect of time and solids loading on Protein Recovery.

	SS	df	MS	F value	p value
**(1) Time**	789.048	1	789.048	19.592	0.047
**(2) Solids loading**	1097.597	1	1097.597	27.253	0.0348
**(1) by (2)**	1142.440	1	1142.440	28.367	0.0335
**Lack of fit**	1241.704	1	1241.704	30.831	0.031
**Pure error**	80.547	2	40.274		
**Total SS**	4351.337	6			

6.2.6 Pareto chart for Protein Recovery:

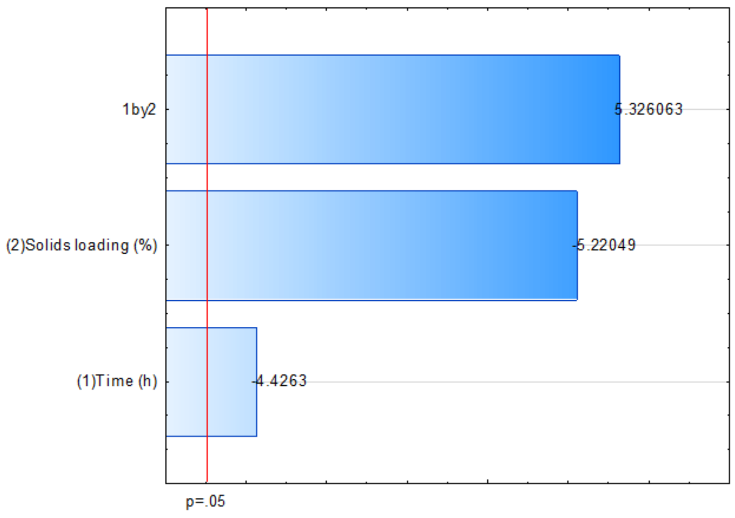

Figure S4. Pareto chart for the effect of time and solids loading on protein removal.

6.2.7 Surface Response

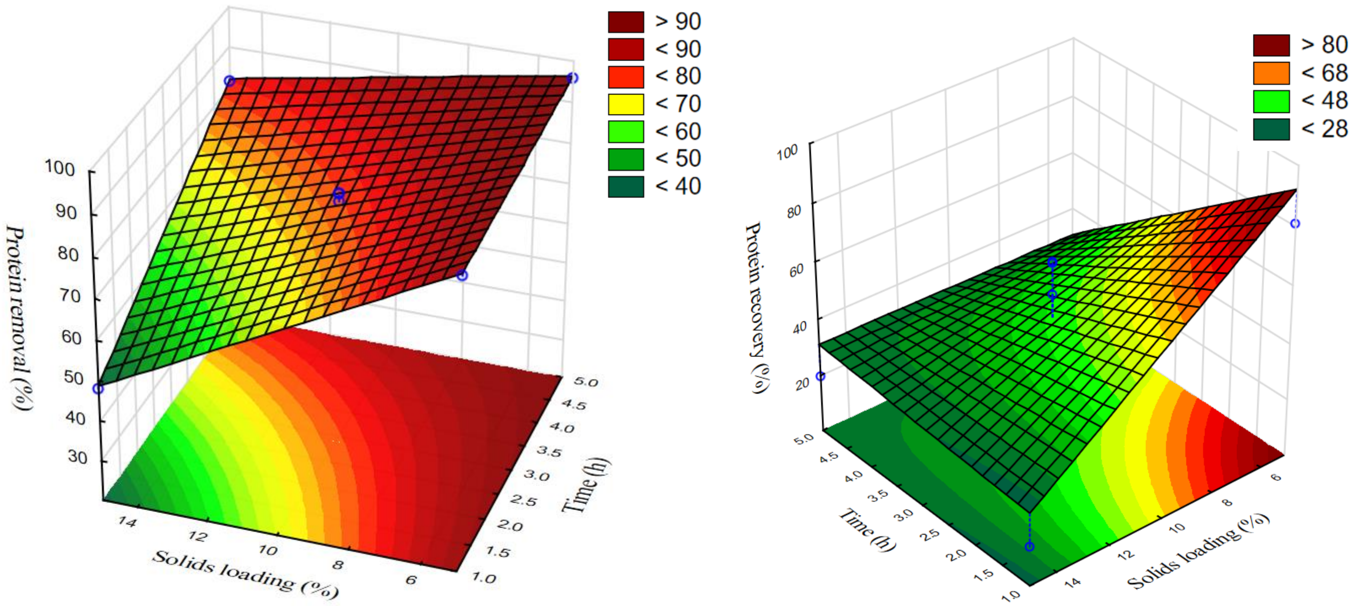

Figure S5. Response Surface Model plots for the (a) protein removal (%) and (b) protein recovery (%) as a function of extraction time (h) and solids loading (%).

6.3 ANOVA: effect of water content of IL on Protein Recovery

Table S7. ANOVA for the effect of water content on Protein Recovery.

	SS	Df	MS	F value	p value
**Intercept**	60809.71	1	60809.71	963.75	0.000000
**Water content**	1254.34	3	418.11	6.63	0.015
**Error**	504.78	8	63.10		

Table S8. Statistical model parameters for the effect of water content on Protein Recovery.

Multiple R	Multiple R^2	Adjusted R^2	SS model	Df model	MS model	SS residual	Df residual	MS residual	F	p
0.844	0.713	0.605	1254.34	3	418.11	504.7	8	63.097	6.63	0.015

6.4 Summary of effects for the 2^2 factorial design with three center point triplicates – Factors: Solids loading and water content

Table S9. Effect estimates, p value and confidence intervals. R^2 = 0.8778; Adjusted R^2: 0.7556

	Effect	Std. error	T(3)	p	Coeff	Std. error Coeff
**Mean/Intercept**	53.74	1.47	36.46	0.00	49.44	1.47
**(1) Time**	-48.11	3.90	-12.34	0.01	-59.50	1.95
**(2) S. loading**	-18.48	3.90	-4.74	0.04	-29.87	1.95
**(1) by (2)**	5.16	3.90	1.32	0.32	-6.23	1.95

Table S10. ANOVA for the effect of time and solids loading on Protein Recovery.

	SS	df	MS	F value	p value
**(1) Solids loading**	2314.575	1	2314.575	152.1556	0.006508
**(2) Water content**	341.577	1	341.577	22.4546	0.041764
**(1) by (2)**	26.576	1	26.576	1.7470	0.317178
**Lack of fit**	343.062	1	343.062	22.5522	0.041595
**Pure error**	30.424	2	15.212		
**Total SS**	3056.213	6			

6.2.6 Pareto chart for Protein Recovery:

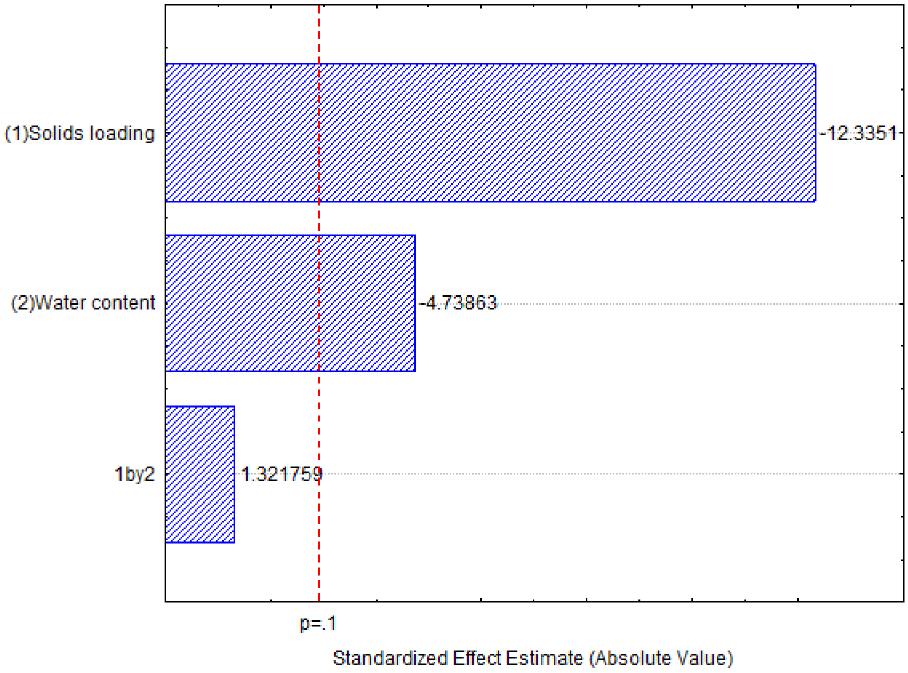

Figure S6. Pareto chart for the effect of time and solids loading on protein removal.

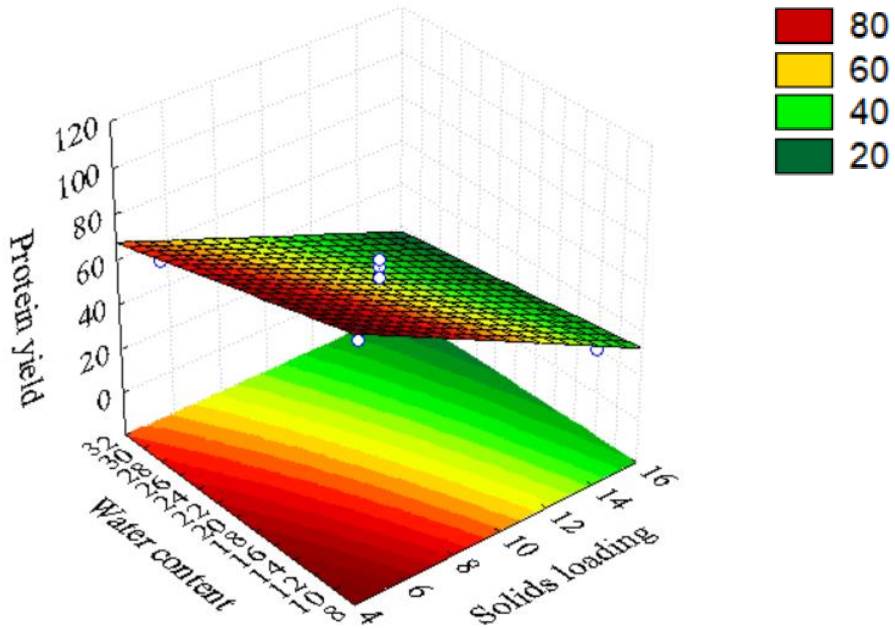

Figure S7. Response Surface Model plots for the protein recovery (%) as a function of water content (%) and solids loading (%).

7 MD simulations – Computation protocol

Force field parameters for the IL and protein were obtained from virtual-site OPLS and OPLS-AA force fields, respectively[9], while the TIP3P water model was employed[10]. Numerical integration was carried out using a Verlet leapfrog algorithm with a 2 fs time step, employing a 1.0 nm cutoff for short-range interactions and particle-mesh Ewald summation for long-range electrostatics[11]. The systems were maintained at 300 K using a modified Berendsen thermostat and at 1 bar pressure with the Parrinello-Rahman algorithm. Subsequently, production simulations were conducted for 20 nanoseconds, incorporating soft harmonic constraints for some structures and cubic boxes for others. The ComplexMixtures.jl software[12,13] was utilized to compute minimum-distance distribution functions, KB integrals, and solute-solvent discrimination parameters[14,15]. A 20 Å cutoff was employed to define the "protein domain," with properties outside this region utilized to evaluate solution characteristics independent of the protein's influence.

Equilibrium molecular dynamics simulations of the structures solvated by water and a variety of IL pairs and concentrations were performed using GROMACS.2018.3 CUDA[16,17]. The initial configurations of the systems were built with Packmol[18,19]. Parameters to describe the ionic liquids and the protein were obtained, respectively, from the virtual-site OPLS[20], and OPLS-AA force fields, and the water model used was the TIP3P model[10]. Numerical integration of the equations of motion was performed using the Verlet leapfrog algorithm with a timestep of 2 fs. A cutoff of 1.0 nm was used for short-range electrostatic and Lennard-jones interactions. The long-range electrostatics were calculated using particle-mesh Ewald[11] summation with a fourth-order interpolation and a grid spacing of 0.16 nm. The temperature of the systems was set to 300 K. The modified Berendsen thermostat was used to keep the temperature fixed with a relaxation time of 0.1 p[21,22].

Using the Parrinello-Rahman algorithm with a relaxation time of 2 ps and isothermal compressibility of 4.5 10-5 bar, the pressure was kept constant at 1 bar[23,24]. Each system's energy was originally minimized for 50000 Steepest-Descent[25] steps while all protein coordinates remained constant.

1 ns of thermal equilibration in the NVT ensemble was followed by 5 ns MD in isothermic-isobaric (NPT) conditions with the protein backbone fixed. The restrictions employed in simulations with the native conformation were removed after these stages, and 1 ns simulations with constant pressure and temperature were undertaken. Production simulations lasted 20 nanoseconds and were run in the NPT ensemble as well. Each structure set consisted of 10 conformations that were simulated separately using the procedure outlined above. We chose to run many brief simulations since the Ubiquitin structure stays closer to the initial state, and we get a good sample of the solvent structure. Soft harmonic constraints with a 10 kJ mol^{-1} $Å^{-2}$ force constant were added to the Cα atoms of the structures, preventing the structures from rotating. This allowed the use of simulation boxes with dimensions adjusted for each conformation. These constraints were not required in the simulations of the folded state because a cubic box was used.

The MDDFs, KB integrals, and discrimination of solute and solvent atomic contributions are computed using the ComplexMixtures.jl[12,13] package (http://m3g.github.io/ComplexMixtures.jl). The density was derived from the average number of minimum distances at each 0.1 Å bin and were used to get the KB integrals and preferential solvation parameters. To declare KB integral convergence, we choose R = 20 Å in all systems (which is unusually large[14,15], demands large solvation boxes, and was required because of the size and electrostatic nature of the IL ions). The solution volume closer to the solute than this distance was therefore considered the "protein domain", i. e. the region of the solution where the solution structure is affected by the presence of the protein. The volume outside this domain contains the mixture of cosolvents and is used to deduce the structure and thermodynamic properties of the solution without the protein (for instance, the effective bulk concentration of the solutions is obtained from this region of the simulation box).

7.1 System build up and the choice of Ubiquitin

Since the squid pen is a biomaterial that contains several proteins, Ubiquitin was selected due to its abundancy, and for being a neutral protein, which simplifies the study of solvation structures in charged cosolvents. The ILs used were formed by a combination of two anions: acetate, $[OAc]^-$, and methane sulfonate, $[MeSO_3]^-$; and two cations: cholinium, $[Ch]^+$, and monoethanolamonium, $[MEA]^+$. The goal of these MD simulations was to understand why protein solubilization was preferential for [Ch][OAc] and not for the other probed ILs, such as [MEA][OAc] sharing the same anion or $[Ch][MeSO_3]$ with the same cation.

Table S11. Reference concentration (RC) of the systems built for the ILs, box sides after system equilibrating, number of components, and water and IL concentration after the system NVT and NPT equilibrating.

**System**	**RC (mol L^{-1})**	**Box sides (Å)**	**Number of water molecules**	**Number of each ion**	**[water]** (mol·L^{-1})	**[IL] (mol L^{-1})**
[Ch][OAc]	3.0	94	14399	1504	26.83 ± 0.03	2.81 ±0.01
$[Ch][MeSO_3]$	3.0	94	14399	1504	28.38 ± 0.01	2.95 ±0.01
[MEA][OAc]	3.0	94	14399	1504	25.30 ± 0.03	2.75 ±0.01

The first line of analysis will be focused on understanding the anion effect on protein solvation by the ILs used. The choline-based IL is said to be the one that provides the more pronounced protein recovery from the squid pen. To investigate the molecular details of the systems, a concentration of 3.0 mol·L^{-1} was used, for being adequate for the computation of distribution functions and KB integrals, as shown in previous studies.

7.2 Supplementary MD results and discussion

The interactions between proteins and other biomolecules with cosolvents are frequently quantified by measuring the preferential solvation parameter. This can be measured using equilibrium dialysis[26] or vapor pressure osmometry[27]. The preferential solvation parameter is related to the variation of the protein chemical potential as the result of the addition of a cosolvent[28]. Another way to interpret this is to express preferential solvation as the change in the cosolvent concentration to keep the chemical potential of the cosolvent constant when the protein is added into the system. Typically, species that establish favourable interactions with the proteins display a positive preferential solvation, leading to protein denaturation and solubilization, such as urea[29].

The Kirkwood-Buff theory of solutions connects the microscopic picture of the solution (the distribution functions) to the macroscopic thermodynamic data. The computational experiments here presented indicate that the IL preferentially solvates the protein when formed by the [OAc] anion. In this accumulation, hydrogen bonds and other polar interactions play a significant role. Since the solution must be electrically neutral at a further distance, the initial interaction between the protein and the anion leads to a net negative charge. This attracts the cations and increases the local IL concentration compared to the bulk IL concentration.

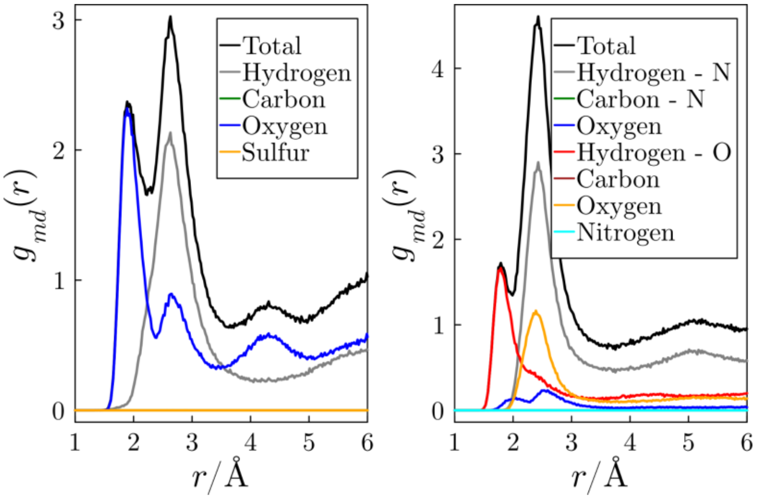

Figure S8. Contributions of different protein residue types to the total MDDF for [$MeSO_3$] (left) and [Ch] (right).

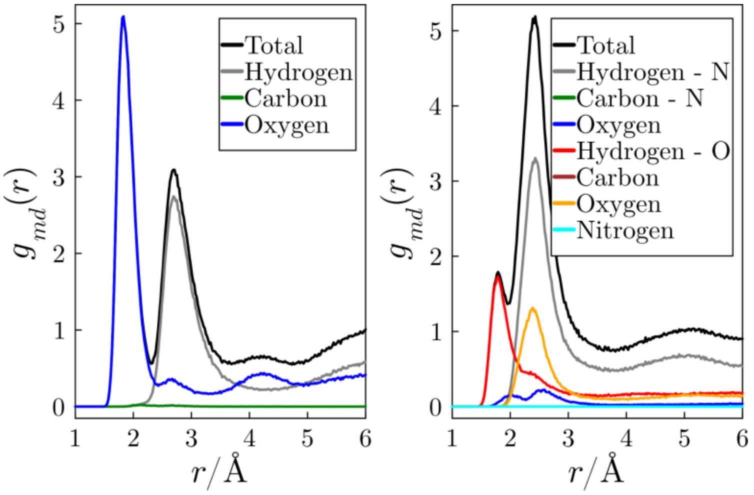

Figure S9. Contributions of different protein residue types to the total MDDF for [OAc] (left) and [Ch] (right).

8 Techno-economic analysis

Operational costs were calculated according to the energy demands obtained in the simulation and normal utility prices. The integrated Aspen Economics package was used for the estimation of the purchased and installed costs of the plant, from which capital and fixed costs were calculated. All the employed parameters for economic estimations can be found in Tables S12-14 (ESI), following previous literature values and typical engineering parameters[30,31]. CO_2 emissions were estimated considering that steam and electricity are generated by burning methane, with thermal efficiencies of 80% and 45%, respectively[32]. The IL price considered was 1.48 $\$\cdot kg^{-1}$ [33]. Feedstock price was considered to be 0.3 $\$\cdot kg^{-1}$, a price previously reported for squid pen powder[34]. Protein was selected as the main product and the minimum selling price of protein was then calculated considering the total annual costs, annual plant depreciation and productivity. For the IL implementation in Aspen Plus v11, a properties package was created. The components were defined as pseudo components, using molecular weight, density, and normal boiling point. COSMOSAC model was selected for the thermodynamical model after generation of the COSMO file following our previous publication with the same IL procedure[35]. The model was modified to use the COSMOSAC-Mathias modification. COSMO-volume was specified as CSACVL component parameter and σ-profile as pure component properties SGPRF1-5.

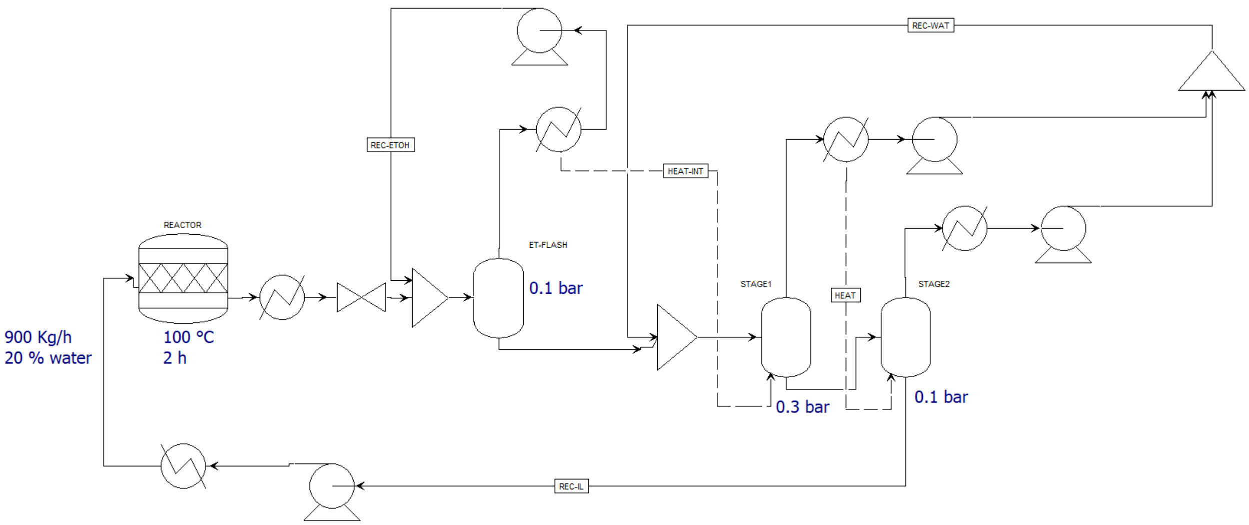

Figure S10. Aspen Plus process flowsheet employed for the simulation of the IL recovery.

Table S12. List of the different utility's prices employed for the economic estimation.

Utility	Price
Steam	0.11 ($/kg)
Cooling water	0.055 ($/m^3)
Electricity	0.3 ($/kWh)

Table S13. List of parameters employed for the total capital costs estimation.

Parameter	Value
Direct field cost (DF)	Total installed costs calculated with Aspen
Direct field labour cost (DFLC)	0.25 x DF
Indirect field cost (IFC)	1.15 x DFLC
Total field cost (TFC)	DF + DFLC + IFC
Home office cost (HOC)	0.3 x DF
Other project cost (OPC)	0.03 x DF + 0.15 x (TFC + HOC)
Total project cost (TPC)	TFC + HOC + OPC

Labour cost (LC) was estimated considering 30 operators with 50.000 $/year of salary.

Table S14. Employed parameters for the estimation of the fixed costs.

Parameter	Value
Maintenance (M)	0.1 x TFC
Laboratory	0.2 x LC
Supervision	0.2 x (LC+M)
Plant overhead	0.5 x (M+LC)
Depreciation	0.1 x (TFC)
Insurance	0.01 x (TFC)
Local taxes	0.02 x (TFC)
Royalties	0.01 (TFC)

9 Single variable experiments

9.4 Extraction time (1 to 4 h)

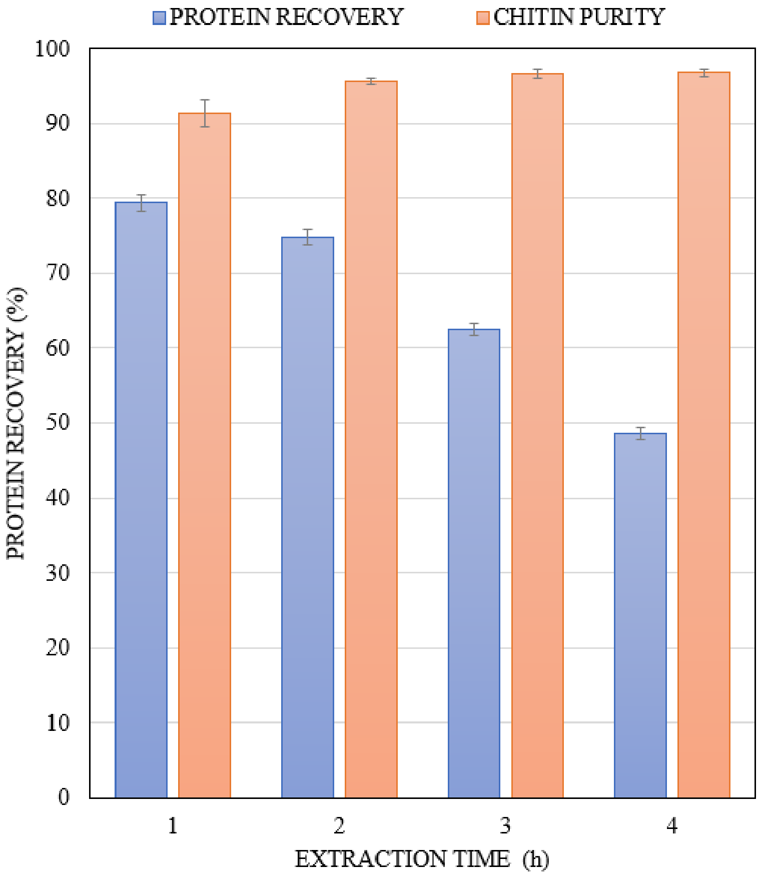

Figure S11. Protein recovery and estimated chitin purity as a function of extraction time with [Ch][OAc] at 100˚C, 20 wt% water content and 5 wt% solids loading.

9.5 Water content

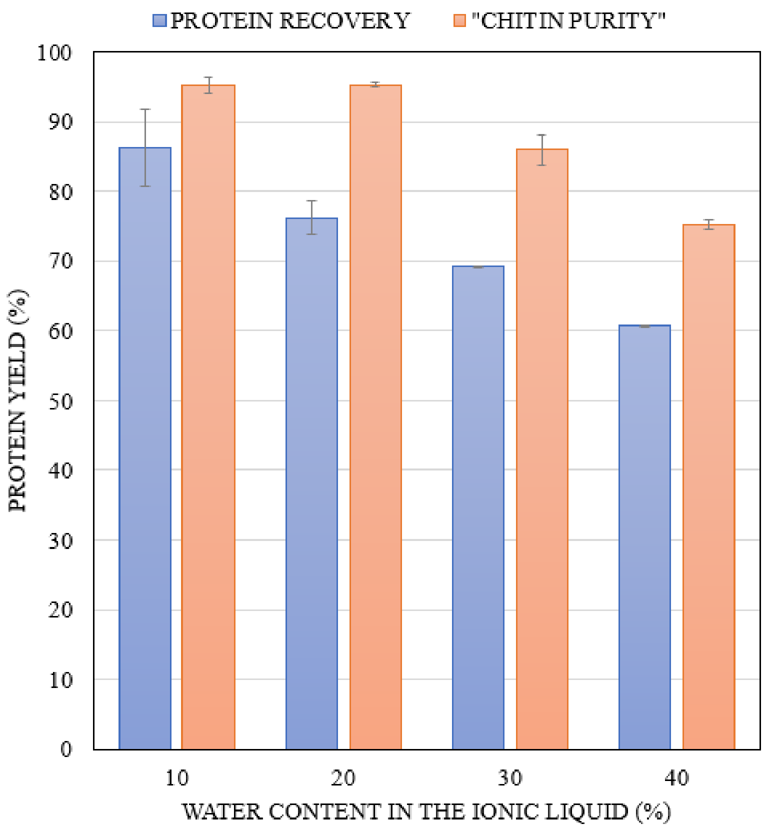

Figure S12. Protein recovery and estimated chitin purity as a function of the water content in [Ch][OAc].

10 IL recycling

Table S15. IL recovery along recycling for three samples of [Ch][OAc].

Cycle	1	2	3	4	5	Average
[Ch][OAc]-1 recovery (%)	97.5	99.0	97.9	97.1	97.1	97.7
[Ch][OAc]-2 recovery (%)	97.1	97.8	98.0	96.7	96.8	97.3
[Ch][OAc]-3 recovery (%)	98.6	98.8	99.0	98.0	98.0	98.5

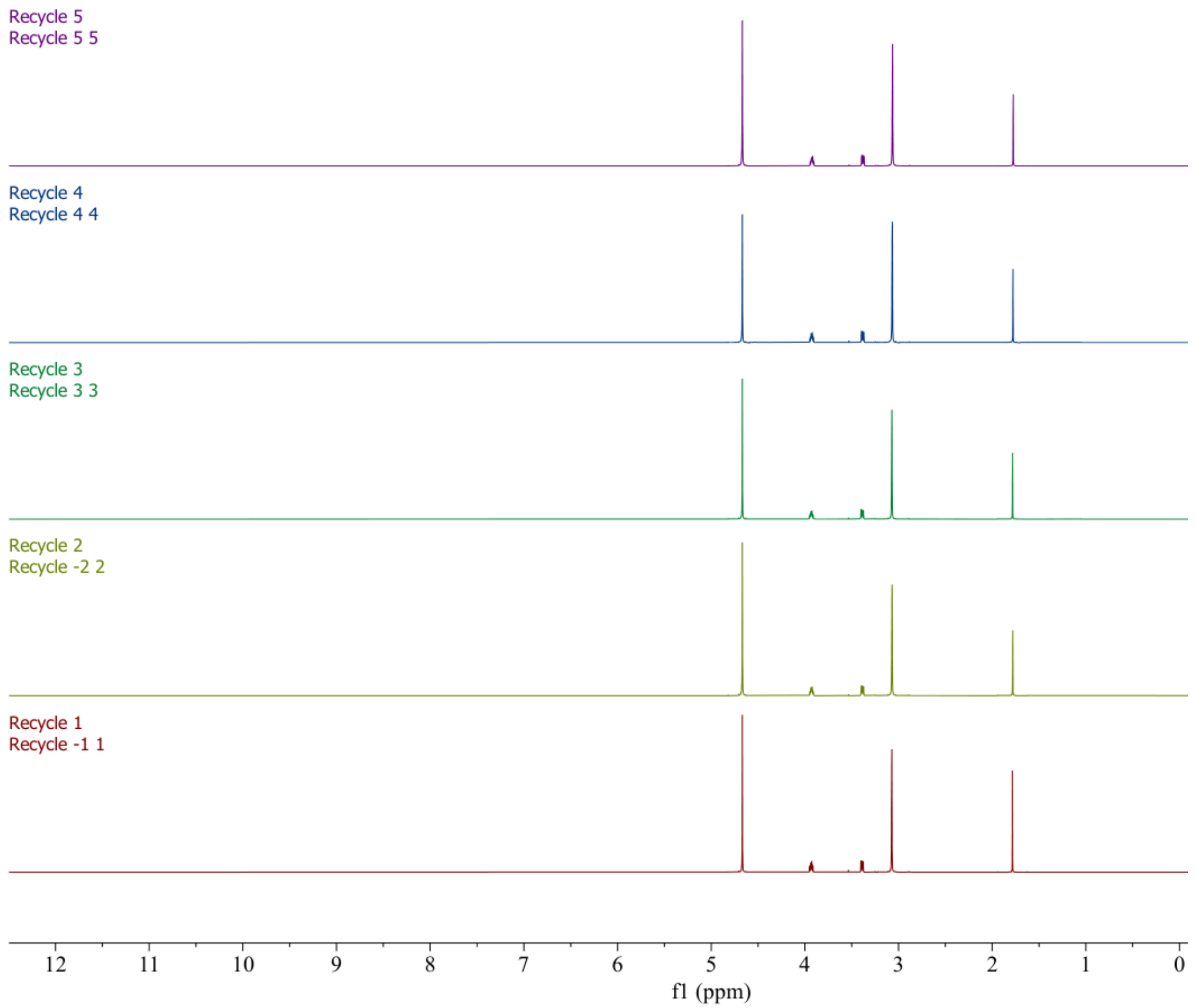

Figure S13. ^{1}H-NMR spectra of [Ch][OAc] along five cycles after each protein extraction.

11 Material analysis

Once the β-chitin and protein were obtained under the optimized conditions with [Ch][OAc] — 100 ˚C, 2 h, 5 wt% solids loading and 20 wt% water — a comprehensive material characterization has been performed to better understand the two streams that were produced.

11.4 ^{1}H-NMR of β-chitin

The ^{1}H-NMR spectrum of the β-chitin was depicted in Figure S9. The estimated degree of acetylation was 94% which basically confirms that the chitin has not been deacetylated by the alkaline [Ch][OAc]. McReynods et al. (2022)[36] employed alkaline DES made of K_2CO_3/glycerol in a molar ratio of 1:5 and observed a decrease in the deacetylation degree from 97% to the conventional method with NaOH to between 77-88% with the DES, which, even though are less alkaline than NaOH, were performed at higher temperatures, 120 ˚C. Shamshina et al. (2023) employed an acidic protic IL, [C_4mim][HSO_4], to produce α-chitin nano whiskers. They observed that the degree of acetylation was maintained after the α-chitin treatment with the IL (around 82%).

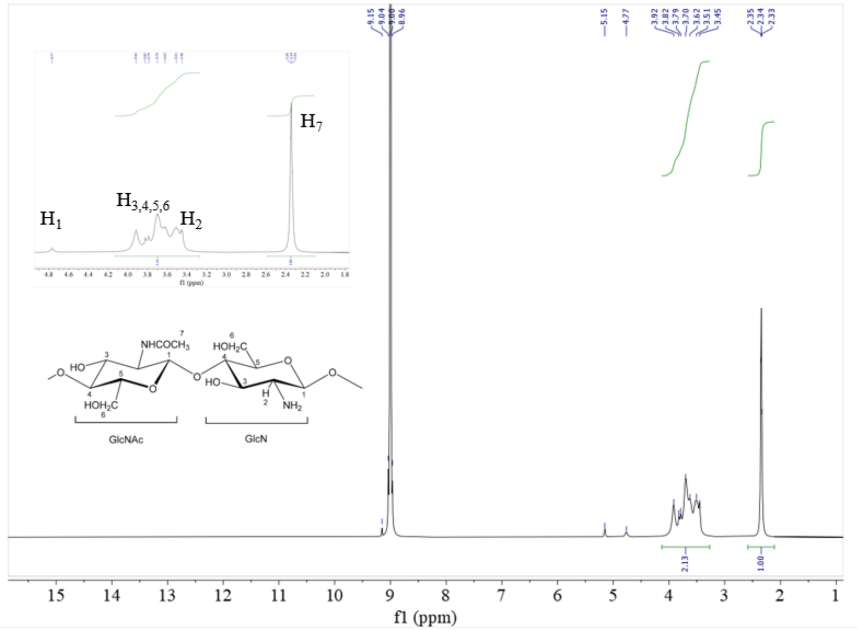

Figure S14. ^{1}H-NMR spectrum of β-chitin in 30 v/v% DCl/D_2O.

11.5 FT-IR

The FT-IR spectra of the extracted fractions, together with the squid pen and a sample of commercial β-chitin were shown in Fig. S8. Several characteristic absorption bands can be seen across the four spectra which are the O–H stretching broad band at 3260 cm^{-1}, the C–H stretching at 2950 and 2860 cm^{-1}, the amide I C=O stretching at 1630 cm^{-1}, the amide II N–H bending at 1511 cm^{-1} and the C–O–C and C–O strechings between 950-1200 cm^{-1} similar to results found elsewhere [37–40]. The main differences across the samples will be related with the intensity of the bands. It can be noted that the protein and the squid pen (which is mostly composed of proteins) present strong absorptions at 1630 and 1511 cm^{-1} related to the amine bands. Whereas with the β-chitin, even though present — chitin contains N-acetyl groups which are amides — such bands are lower in intensity. On the other hand, due to the high number of C–O modes of vibration, squid pens and both β-chitins present strong absorptions between 950-1200 cm^{-1}, which, conversely, are less pronounced in the protein sample. No major differences were observed amongst the β-chitin and protein samples obtained from the screening and optimisation steps.

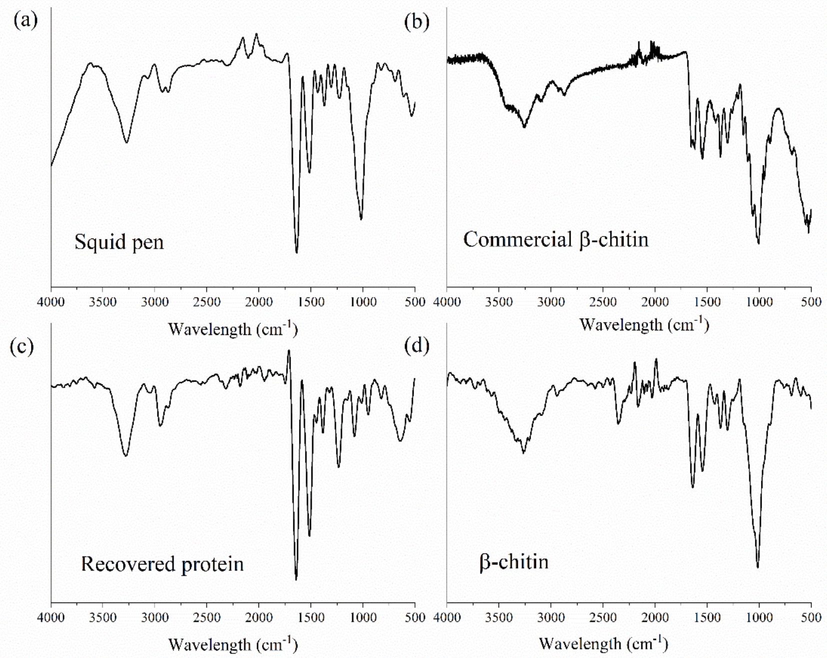

Figure S15. FT-IR spectra of (a) Squid pen powder; (b) commercial β-chitin; (c) recovered protein extracted with [Ch][OAc]; (d) β-chitin from [Ch][OAc] extraction. Reaction conditions were: 100 ℃, 2h, 5wt% solids loading and 20 wt% water content.

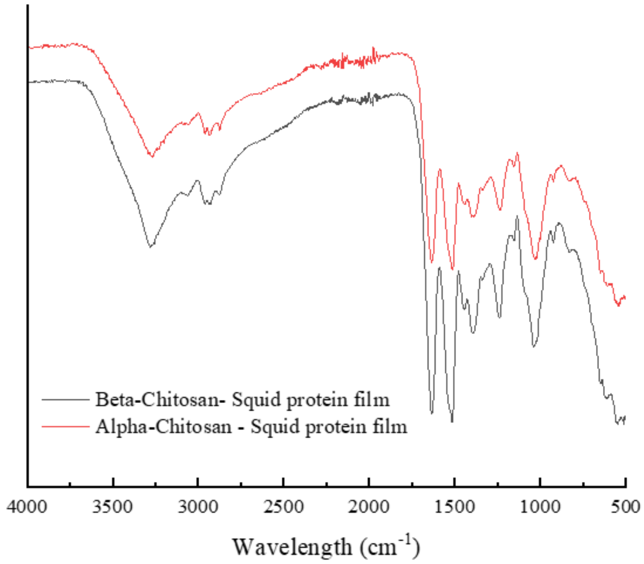

Figure S16. FT-IR spectra of the biocomposite films of α or β chitosan with squid pen protein and glycerol (6.7/3.3/1.0 mass ratios).

11.6 TGA

The TGA and DTG of the squid pen and the obtained fractions, β-chitin and protein were shown in Fig. X (ESI). A trend can be noticed in which, similarly to the FT-IR spectra, the squid pen had intermediary behaviour between their two components where T_{onset} for the squid pen was 318 ℃, for the β-chitin it was 326 ℃, and for the squid protein 276 ℃. Additionally, the TGA pattern for squid pen resembled more of the protein since it's the major component. Since proteins are more sensitive to high temperatures, it was expected to have a lower T_{onset} than the β-chitin. The DTGs also showed the observed trend with the max DTG values for squid pen, β-chitin and protein of 355℃, 372℃ and 333℃ respectively. Similar trends were observed by Kumari and Kishor (2020) [41] on β-chitin from cuttlefish, Cabrera-Varjas et al. (2021) [39] on β-chitin from Humboldt squid and McReynolds et al. (2022) [36] on the β-chitin extracted from loligo vulgaris.

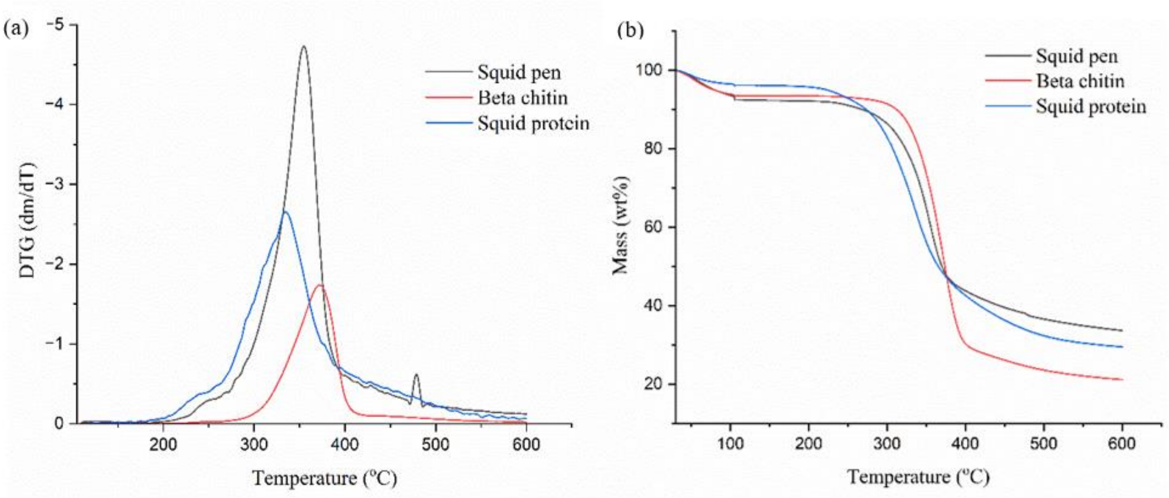

Figure S17. TGA (a) and DTG (b) of squid pen, β-chitin and protein obtained from [Ch][OAc] extraction. Reaction conditions were: 100 ℃, 2h, 5wt% solids loading and 20 wt% water content.

11.7 XRD

The crystalline structure of the β- chitin obtained from the [Ch][OAc] extraction was analyzed by x- ray diffraction together with the squid pen for comparison. The corresponding diffractograms were shown in Fig. X (ESI). The diffractogram of both samples present one peak at 2θ = 8.63° and 9.22° for β-chitin and squid pen, and another more intense peak at 2θ = 19.91° and 19.61°for the β-chitin and squid pen. These

peaks correspond to reflection of the crystal planes [010] and [110][36,39]. The crystallinity index of β-chitin and squid pen were 82% and 70% which shows that removal of the more amorphous protein leaves a highly crystalline β-chitin. Cuong et al. (2016) [37] observed that alkaline extraction (NaOH, 80℃, 10h) of the squid pen from Loligo chenisis increased the CrI from 52 to 62%. A CrI of 70% has been observed on the β-chitin obtained from a two-step acidic (1M HCl, 2h) and alkaline extraction (1M NaOH, 3h) of the Humboldt squid [39]. From the literature values, it seems like the CrI from [Ch][OAc] extraction was higher than the conventional one with NaOH. McReynods et al. (2022)[36] also found the same behaviour while exploring the use of DES made of K_2CO_3/glycerol in a molar ratio of 1:5. They managed to obtain β-chitins with CrI between 88-91% and argued that a possible reason for higher values compared to the alkaline extraction could be a slight deacetylation of chitin, decreasing the amount of N-acetylglucosamine that forms the crystal structure.

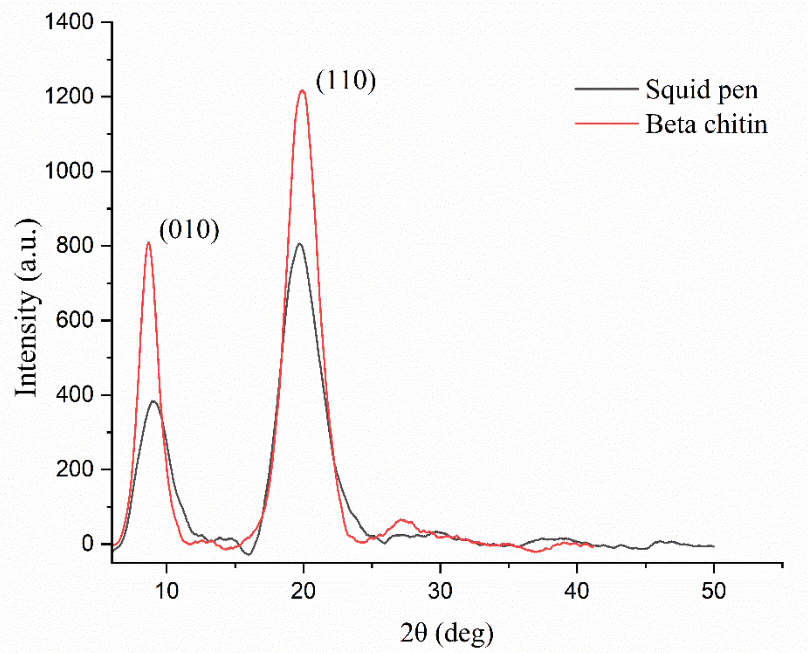

Figure S18. X-ray diffratogram of β-chitin (in red) and squid pen powder (in black).

11.8 SDS-PAGE

The electrophoretic profile of some of the squid proteins obtained in this study were depicted in Fig. 10. It can be seen that the proteins presented a similar electrophoretic pattern with two distinct bands at 10 kDa and another one between 15 and 25 kDa. Messerli et al. (2019) [42] studied the composition of the squid pen of *Doryteuthis pealeii* and found that the majority of the proteins were actin, myosin and tropomyosin together with a number of intracellular proteins. However, their SDS-PAGE profile showed bands below 10 kDa and above 40 kDa, which may indicate that either the proteins from loligo sp from this work were different, or they have been degraded during the extraction. Omote-Sibina and Roldan-Acero (2023) [6] obtained an alkaline hydrolysate from giant squid (Dosidicus gigas) and observed protein bands that they attributed to myosin (between 15 and 21 kDa), troponin (between 21 and 31 kDa) and tropomyosin (between 31 and 45 kDa). Therefore, the proteins band are likely to be related to these muscular proteins from squid and presented low molecular weight.

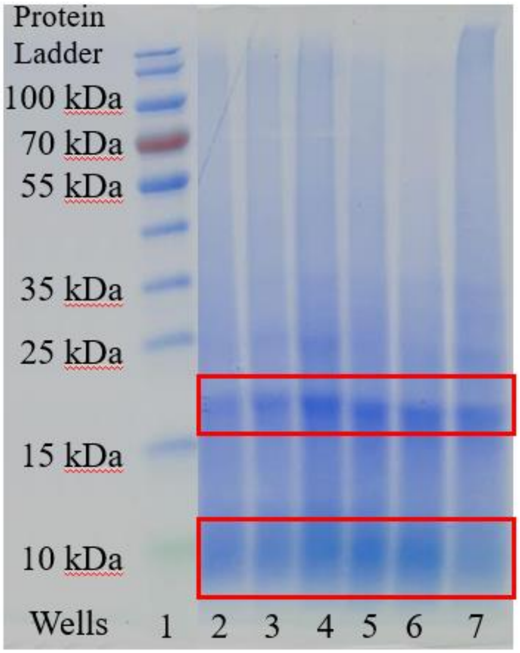

Figure S19. SDS-PAGE of squid protein samples. (1) Protein ladder (2) [Ch][OAc] screening (3) PT OPT 1 (100°C, 5h, 15% solids) (4) PT OPT 2 (100°C, 5h, 5% solids) (5) PT OPT 5 (100°C, 1hr, 15% solids) (6) PT OPT 6 (100°C, 1hr, 5% solids) (7) PT from recycle (100°C, 2hr, 5% solids).

11.9 Amino acid profile

The elemental analysis of the β-chitin and protein showed a nitrogen content of 7.60% and 14.4% which corresponds to β-chitin purity of 95.6% and a protein purity of 90% respectively. These values place the [Ch][OAc] fractionation amongst the best ones to produce high purity streams from squid waste. The amino acid profile of the squid pen protein was shown in Table S15. The total amino acid content is lower than the purity estimated by CHN analysis, however, it is important to address that amino acid profiling generally underestimate the protein content because it is based on an acidic hydrolysis. Also, tryptophan has not been quantified on the analysis.

Amino acids serve as the building blocks for protein synthesis in the diets of fish, prawns, and other aquatic animals. Once synthesised, proteins play a variety of roles in the body, including building muscular tissue and catalysing biological events (enzymes), among other things [43]. Animals receiving low-quality protein have a detrimental effect on the environment and aquaculture's profitability. Ten of the twenty amino acids found in nature are thought to be essential for fish and other aquatic species' diets. They are histidine, valine, phenylalanine, threonine, tryptophan, lysine, methionine, isoleucine, leucine, and arginine [44]. They must be obtained through diet since their bodies cannot synthesise them, or cannot synthesise them in sufficient amounts. Tyrosine and cysteine are not classified as essential amino acids (EAAs) in traditional animal nutrition textbooks, as the liver can produce them from methionine and phenylalanine, respectively. However, the inability of all animals to form the carbon skeletons for methionine and phenylalanine means that they cannot be synthesised again [45]. The squid protein concentrate obtained in this work had an overall content of 43% excluding tryptophan and this value reaches up to 56% if cysteine and tyrosine are included. The major essential amino acids were histidine, leucine and valine, whereas tyrosine, proline and alanine made up the majority of non-essential amino acids. Youn et al. (2013) [46] observed that the squid pen from *Todarodes pacifica* presented high tyrosine and histidine content in their amino acid profile, which is typical of globular proteins and also related to the presence of chitin binding domains in such proteins [47]. It is important to stress that differences in the amino acid profile varies due to variations in species, as well life

strategies (benthic, nektobenthic, benthopelagic and pelagic) and even habitats (neritic, demersal, oceanic and deep sea)[48]

Table S16. Amino acid composition of the squid pen protein concentrate extracted with [Ch][OAc].

**Amino acid**	**%**	Essentials	Essentials plus cys+Tyr
Cystine	2.01		2.01
Aspartic	6.97		
Methionine	0.88	0.88	0.88
Threonine	3.45	3.45	3.45
Serine	2.51		
Glutamic	3.89		
Glycine	6.31		
Alanine	7.28		
Valine	5.31	5.31	5.31
Iso-Leucine	2.32	2.32	2.32
Leucine	6.71	6.71	6.71
Tyrosine	7.99		7.99
Phenylalanine	2.52	2.52	2.52
Histidine	8.86	8.86	8.86
Lysine	3.06	3.06	3.06
Arginine	1.37	1.37	1.37
Proline	8.55		
Total	80	43[a]	56[a]

[a] Relative to the total amino acid content.

12 Acid-base ratio of ILs

Table S17. Acid-base ratios of the ILs estimated by ^{1}H-NMR.

**IL**	**Acid-base ratio**
[Ch][OAc]	0.95
[Ch][$MeSO_3$]	1.07
[Ch][HSO_4]	0.97
[MEA][OAc]	0.97
[MEA][$MeSO_3$]	1
[MEA][HSO_4]	0.99

13 References

1. Nakasu, P.Y.S., Clarke, C.J., Rabelo, S.C., Costa, A.C., Brandt-Talbot, A., and Hallett, J.P. (2020). Interplay of acid–base ratio and recycling on the pretreatment performance of the protic ionic liquid monoethanolammonium acetate. ACS Sustain. Chem. Eng. *8*, 7952–7961. 10.1021/acssuschemeng.0c01311.

2. Pérez-Álvarez, L., Ruiz-Rubio, L., and Vilas-Vilela, J.L. (2018). Determining the deacetylation degree of chitosan: opportunities to learn instrumental techniques. J. Chem. Educ. *95*, 1022–1028. 10.1021/acs.jchemed.7b00902.

3. Polesca, C., Passos, H., Neves, B.M., Coutinho, J.A.P., and Freire, M.G. (2023). Valorization of chicken feathers using aqueous solutions of ionic liquids. Green Chem. *25*, 1424–1434. 10.1039/D2GC04477C.

4. Hames, B., Sluiter, A., and Scarlata, C. (2008). Determination of protein content in biomass. Laboratory Analytical Procedure (LAP) (National Renewable Energy Laboratory).

5. Gosukonda, V., Singh, H., and Gosukonda, R. (2020). Comparative analysis of nitrogen-to-protein conversion factors for determining net protein content in six superfoods. JMBFS *9*, 856–860. 10.15414/jmbfs.2020.9.4.856-860.

6. Omote-Sibina, J.R., and Roldán-Acero, D.J. (2023). Identification of soluble proteins present in giant squid (Dosidicus gigas) meal for human consumption. Agron. Mesoam., 50264. 10.15517/am.v34i2.50264.

7. Nurjanah (2012). Proximate, Nutrient and Mineral Composition of Cuttlefish (Sepia recurvirostra). AJFST *4*, 220–224.

8. Berger, L.R.R., Stamford, T.C.M., Stamford-Arnaud, T.M., de Alcântara, S.R.C., da Silva, A.C., da Silva, A.M., do Nascimento, A.E., and de Campos-Takaki, G.M. (2014). Green conversion of agroindustrial wastes into chitin and chitosan by Rhizopus arrhizus and Cunninghamella elegans strains. Int. J. Mol. Sci. *15*, 9082–9102. 10.3390/ijms15059082.

9. Robertson, M.J., Tirado-Rives, J., and Jorgensen, W.L. (2015). Improved Peptide and Protein Torsional Energetics with the OPLSAA Force Field. J. Chem. Theory Comput. *11*, 3499–3509. 10.1021/acs.jctc.5b00356.

10. Jorgensen, W.L., Chandrasekhar, J., Madura, J.D., Impey, R.W., and Klein, M.L. (1983). Comparison of simple potential functions for simulating liquid water. J. Chem. Phys. *79*, 926. 10.1063/1.445869.

11. Darden, T., York, D., and Pedersen, L. (1993). Particle mesh Ewald: An N· log(N) method for Ewald sums in large systems. J. Chem. Phys. *98*, 10089. 10.1063/1.464397.

12. Bezanson, J., Edelman, A., Karpinski, S., and Shah, V.B. (2017). Julia: A fresh approach to numerical computing. SIAM Rev. *59*. 10.1137/141000671.

13. Martínez, L. (2022). ComplexMixtures.jl: Investigating the structure of solutions of complex-shaped molecules from a solvent-shell perspective. J. Mol. Liq. *347*, 117945. 10.1016/j.molliq.2021.117945.

14. Baynes, B.M., and Trout, B.L. (2003). Proteins in Mixed Solvents: A Molecular-Level Perspective. J. Phys. Chem. B *107*, 14058–14067. 10.1021/jp0363996.

15. Martínez, L., and Shimizu, S. (2017). Molecular Interpretation of Preferential Interactions in Protein Solvation: A Solvent-Shell Perspective by Means of Minimum-Distance Distribution Functions. J. Chem. Theory Comput. *13*, 6358–6372. 10.1021/acs.jctc.7b00599.

16. Van Der Spoel, D., Lindahl, E., Hess, B., Groenhof, G., Mark, A.E., and Berendsen, H.J.C. (2005). GROMACS: fast, flexible, and free. J. Comput. Chem. *26*, 1701–1718. 10.1002/jcc.20291.

17. Kohnke, B., Ullmann, R.T., Kutzner, C., Beckmann, A., Haensel, D., Kabadshow, I., Dachsel, H., Hess, B., and Grubmüller, H. (2017). A Flexible, GPU - Powered Fast Multipole Method for Realistic Biomolecular Simulations in Gromacs. Biophys. J. *112*, 448a. 10.1016/j.bpj.2016.11.2402.

18. Martínez, L., Andrade, R., Birgin, E.G., and Martínez, J.M. (2009). PACKMOL: a package for building initial configurations for molecular dynamics simulations. J. Comput. Chem. *30*, 2157–2164. 10.1002/jcc.21224.

19. Martínez, J.M., and Martínez, L. (2003). Packing optimization for automated generation of complex system's initial configurations for molecular dynamics and docking. J. Comput. Chem. *24*, 819–825. 10.1002/jcc.10216.

20. Doherty, B., Zhong, X., and Acevedo, O. (2018). Virtual Site OPLS Force Field for Imidazolium-Based Ionic Liquids. J. Phys. Chem. B *122*, 2962–2974. 10.1021/acs.jpcb.7b11996.

21. Bussi, G., Donadio, D., and Parrinello, M. (2007). Canonical sampling through velocity rescaling. J. Chem. Phys. *126*, 014101. 10.1063/1.2408420.

22. Berendsen, H.J.C., Postma, J.P.M., van Gunsteren, W.F., DiNola, A., and Haak, J.R. (1984). Molecular dynamics with coupling to an external bath. J. Chem. Phys. *81*, 3684. 10.1063/1.448118.

23. Parrinello, M. (1981). Polymorphic transitions in single crystals: A new molecular dynamics method. J. Appl. Phys. *52*, 7182. 10.1063/1.328693.

24. Parrinello, M., and Rahman, A. (1982). Strain fluctuations and elastic constants. J. Chem. Phys. *76*, 2662–2666. 10.1063/1.443248.

25. McSherry, T. (1976). A general steepest descent algorithm. IEEE Trans. Aerosp. Electron. Syst. *AES-12*, 12–22. 10.1109/TAES.1976.308210.

26. Ziv, G., and Haran, G. (2009). Protein folding, protein collapse, and tanford's transfer model: lessons from single-molecule FRET. J. Am. Chem. Soc. *131*, 2942–2947. 10.1021/ja808305u.

27. Courtenay, E.S., Capp, M.W., Anderson, C.F., and Record, M.T. (2000). Vapor pressure osmometry studies of osmolyte-protein interactions: implications for the action of osmoprotectants in vivo and for the interpretation of "osmotic stress" experiments in vitro. Biochemistry *39*, 4455–4471. 10.1021/bi992887l.

28. Canchi, D.R., and García, A.E. (2013). Cosolvent effects on protein stability. Annu. Rev. Phys. Chem. *64*, 273–293. 10.1146/annurev-physchem-040412-110156.

29. Canchi, D.R., Paschek, D., and García, A.E. (2010). Equilibrium study of protein denaturation by urea. J. Am. Chem. Soc. *132*, 2338–2344. 10.1021/ja909348c.

30. Polesca, C., Al Ghatta, A., Passos, H., Coutinho, J.A.P., Hallett, J.P., and Freire, M.G. (2023). Sustainable keratin recovery process using a bio-based ionic liquid aqueous solution and its techno-economic assessment. Green Chem. *25*, 3995–4003. 10.1039/D3GC00850A.

31. Shabani, M.R., and Yekta, R.B. (2006). Chemical processes equipment cost estimation using parametric models. Cost Engineering *48*, 22–25.

32. Al Ghatta, A., Wilton-Ely, J.D.E.T., and Hallett, J.P. (2021). From sugars to FDCA: a techno-economic assessment using a design concept based on solvent selection and carbon dioxide emissions. Green Chem. 10.1039/D0GC03991H.

33. Ovejero-Pérez, A., Ayuso, M., Rigual, V., Domínguez, J.C., García, J., Alonso, M.V., Oliet, M., and Rodriguez, F. (2021). Technoeconomic Assessment of a Biomass Pretreatment + Ionic Liquid Recovery Process with Aprotic and Choline Derived Ionic Liquids. ACS Sustain. Chem. Eng. *9*, 8467–8476. 10.1021/acssuschemeng.1c01361.

34. Wang, S.-L., Wang, C.-Y., Yen, Y.-H., Liang, T.-W., Chen, S.-Y., and Chen, C.-H. (2012). Enhanced production of insecticidal prodigiosin from Serratia marcescens TKU011 in media containing squid pen. Process Biochemistry *47*, 1684–1690. 10.1016/j.procbio.2011.07.010.

35. Abouelela, A.R., Al Ghatta, A., Verdía, P., Shan Koo, M., Lemus, J., and Hallett, J.P. (2021). Evaluating the Role of Water as a Cosolvent and an Antisolvent in [HSO_4]-Based Protic Ionic Liquid Pretreatment. ACS Sustain. Chem. Eng. *9*, 10524–10536. 10.1021/acssuschemeng.1c02299.

36. McReynolds, C., Adrien, A., de Fraissinette, N.B., Olza, S., and Fernandes, S.C.M. (2022). Deep eutectic solvents for the extraction of β-chitin from Loligo vulgaris squid pens: a sustainable way to valorize fishery by-products. Biomass Conv. Bioref. 10.1007/s13399-022-03569-9.

37. Cuong, H.N., Minh, N.C., Van Hoa, N., and Trung, T.S. (2016). Preparation and characterization of high purity β-chitin from squid pens (Loligo chenisis). Int. J. Biol. Macromol. *93*, 442–447. 10.1016/j.ijbiomac.2016.08.085.

38. Susana Cortizo, M., Berghoff, C.F., and Alessandrini, J.L. (2008). Characterization of chitin from Illex argentinus squid pen. Carbohydr. Polym. *74*, 10–15. 10.1016/j.carbpol.2008.01.004.

39. Cabrera-Barjas, G., González, C., Nesic, A., Marrugo, K.P., Gómez, O., Delattre, C., Valdes, O., Yin, H., Bravo, G., and Cea, J. (2021). Utilization of Marine Waste to Obtain β-Chitin Nanofibers and Films from Giant Humboldt Squid Dosidicus gigas. Mar. Drugs *19*. 10.3390/md19040184.

40. Balitaan, J.N.I., Martin, G.A.V., and Santiago, K.S. (2021). Revamping squid gladii to biodegradable composites: In situ grafting of polyaniline to β-chitin and their antibacterial activity. J. Bioact. Compat. Polym. *36*, 13–28. 10.1177/0883911520973239.

41. Kumari, S., and Kishor, R. (2020). Chitin and chitosan: origin, properties, and applications. In Handbook of chitin and chitosan (Elsevier), pp. 1–33. 10.1016/B978-0-12-817970-3.00001-8.

42. Messerli, M.A., Raihan, M.J., Kobylkevich, B.M., Benson, A.C., Bruening, K.S., Shribak, M., Rosenthal, J.J.C., and Sohn, J.J. (2019). Construction and Composition of the Squid Pen from Doryteuthis pealeii. Biol. Bull. *237*, 1–15. 10.1086/704209.

43. Wu, G., Bazer, F.W., Dai, Z., Li, D., Wang, J., and Wu, Z. (2014). Amino acid nutrition in animals: protein synthesis and beyond. Annu. Rev. Anim. Biosci. *2*, 387–417. 10.1146/annurev-animal-022513-114113.

44. Xing, S., Liang, X., Zhang, X., Oliva- Teles, A., Peres, H., Li, M., Wang, H., Mai, K., Kaushik, S.J., and Xue, M. (2023). Essential amino acid requirements of fish and crustaceans, a meta-analysis. Rev. Aquacult. 10.1111/raq.12886.

45. Wu, G. (2014). Dietary requirements of synthesizable amino acids by animals: a paradigm shift in protein nutrition. J. Anim. Sci. Biotechnol. *5*, 34. 10.1186/2049-1891-5-34.

46. Youn, D.K., No, H.K., and Prinyawiwatkul, W. (2013). Preparation and characteristics of squid pen β- chitin prepared under optimal deproteinisation and demineralisation condition. Int. J. Food Sci. Technol. *48*, 571–577. 10.1111/ijfs.12001.

47. Montroni, D., Sparla, F., Fermani, S., and Falini, G. (2021). Influence of proteins on mechanical properties of a natural chitin-protein composite. Acta Biomater. *120,* 81–90. 10.1016/j.actbio.2020.04.039.

48. Rosa, R., Pereira, J., and Nunes, M.L. (2005). Biochemical composition of cephalopods with different life strategies, with special reference to a giant squid, Architeuthis sp. Mar. Biol. *146*, 739–751. 10.1007/s00227-004-1477-5.
